# Two Gracilioethers Containing a [2(5H)-Furanylidene]ethanoate Moiety and 9,10-Dihydroplakortone G: New Polyketides from the Caribbean Marine Sponge *Plakortis halichondrioides*

**DOI:** 10.3390/app14010281

**Published:** 2023-12-28

**Authors:** Luis A. Amador, Abimael D. Rodríguez, Lesly Carmona-Sarabia, Emilee E Colón-Lorenzo, Adelfa E. Serrano

**Affiliations:** 1 Molecular Sciences Research Center, University of Puerto Rico, 1390 Ponce de León Avenue, San Juan 00926, Puerto Rico; 2 Department of Microbiology and Medical Zoology, University of Puerto Rico School of Medicine, San Juan 00921, Puerto Rico

**Keywords:** *Plakortis halichondrioides*, polyketide, gracilioether, marine sponges

## Abstract

Gracilioether M (**6**) and 11,12-dihydrogracilioether M (**7**), two polyketides with a [2(5H)-furanylidene]ethanoate moiety, along with known plakortone G (**9**) and its new naturally occurring derivative 9,10-dihydroplakortone G (**8**), were isolated from the Caribbean marine sponge *Plakortis halichondrioides*. The structures and absolute configuration of **6**, **7**, and **8** were characterized by analysis of HRESIMS and NMR spectroscopic data, chemical derivatization, and side-by-side comparisons with published NMR data of related analogs. Compounds **6** and **7** and a mixture of **8** and **9** were evaluated for cytotoxicity against MCF-7 human breast cancer cells. In addition, the in vitro antiplasmodial activity against *Plasmodium berghei* of these compounds was scrutinized using a drug luminescence assay.

## 1. Introduction

Natural marine products have risen in prominence as a valuable source of rare secondary metabolites, drawing the attention of scientists and researchers worldwide. These compounds, often extracted from marine organisms such as corals, sponges, mollusks, and algae, are renowned for their remarkable chemical diversity. Research conducted in a plethora of marine ecosystems throughout the world has led to the emergence of a diverse spectrum of molecules possessing an extensive array of biological activities that hold great promise for strengthening medicine and advancing drug discovery [1–3]. In as much as many of these secondary metabolites have displayed potent antifungal, antibacterial, antiviral, and antimalarial properties [4,5], their importance in fighting infectious diseases cannot be overstated. These unique compounds also offer promising pathways toward crafting innovative pharmaceuticals that address the growing challenges of drug-resistant pathogens [6]. Furthermore, the promise of marine natural products in tackling malaria has stirred significant excitement, given their capacity to impede the reproduction of malaria-causing parasites. This brings renewed hope in the battle against this highly destructive illness [7–9].

Marine sponges stand out as a remarkable reservoir of potential lead compounds in the continuous search for novel drugs. Their unique chemical compositions and the diversity of the bioactive molecules that can be obtained from them make these animals a valuable source of inspiration and innovation in the fields of medicinal chemistry and drug discovery [4,5]. Among these interesting organisms, members of the genus *Plakortis* have recently taken the spotlight given their well-known capacity to yield polyketide natural products with a remarkable structural diversity and widespread bioactivity [10–12]. Despite their potential, only a small number of marine polyketide-derived drugs are currently considered to be viable for medical therapeutic use due to their restricted accessibility [12]. Notwithstanding this, marine polyketides have become a focal point of intense research, and investigators throughout the world are diligently exploring the molecular mechanisms behind their biological properties while aiming to harness their therapeutic potential for the benefit of medical science and human health [13–16].

**Figure 1.**
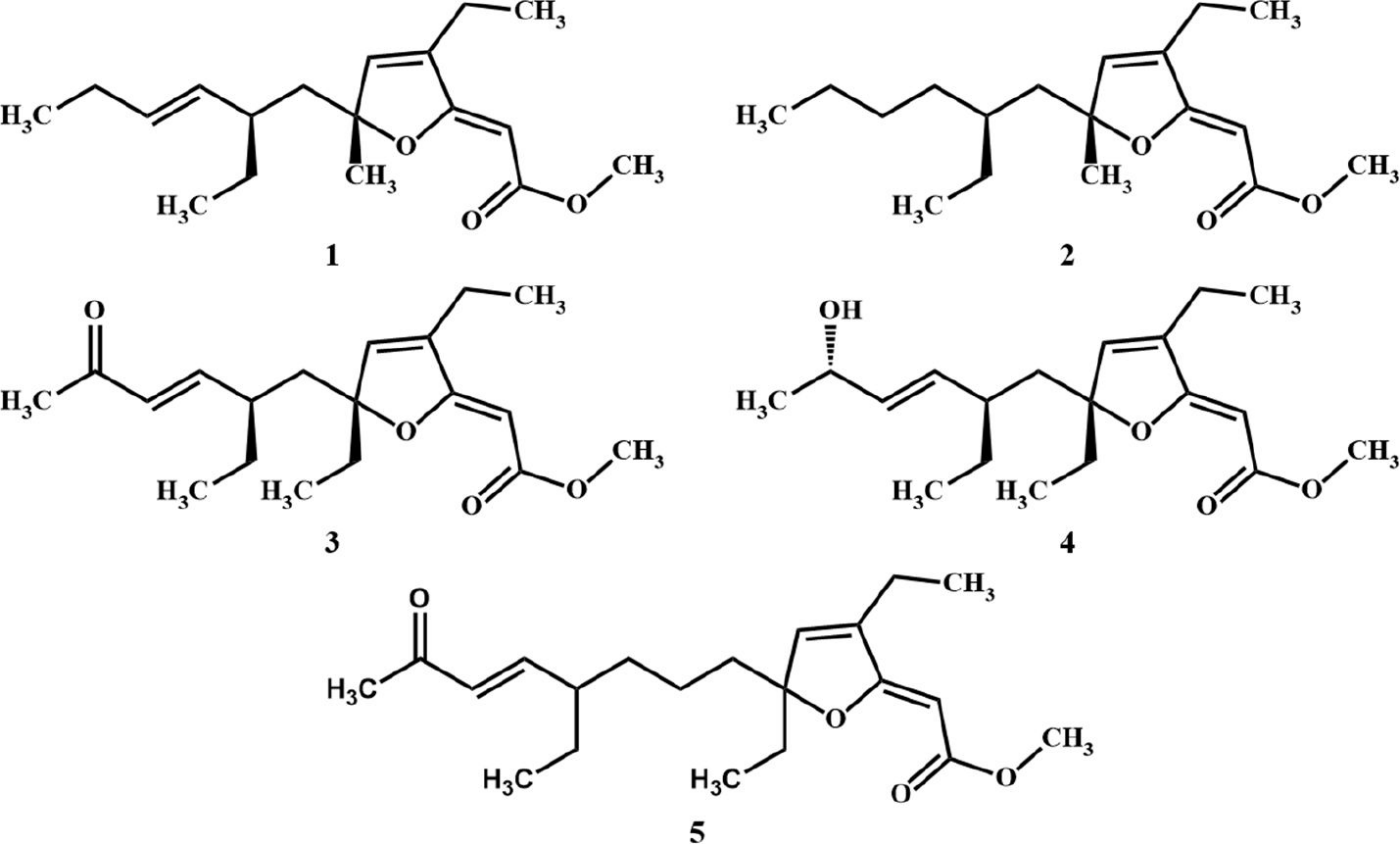
Chemical structures of the marine sponge-derived polyketides **1–5**.

In recent years, several polyketides possessing a [2(5H)-furanylidene]ethanoate (furanylidene) motif have been detected, extracted, and isolated from a wide variety of marine sponges, including species belonging to the genera *Agelas* and *Plakortis*. More specifically, related compounds that were previously isolated from *Plakortis* sponges have been shown to exhibit antimalarial and cytotoxic activities. This category of compounds includes the likes of spongosoritin A (**1**) [17], 9,10-dihydrospongosoritin A (**2**) [18], gracilioether B (**3**) [19], gracilioether C (**4**) [19], and gracilioether L (**5**) [20]. These compounds highlight the prevalence and significance of the furanylidene moiety in polyketides sourced from marine sponges and open new doors to further explore the biological functions and potential applications of these metabolites [21]. Figure 1 provides a visual representation of the chemical structures of compounds **1–5**.

**Figure 2.**
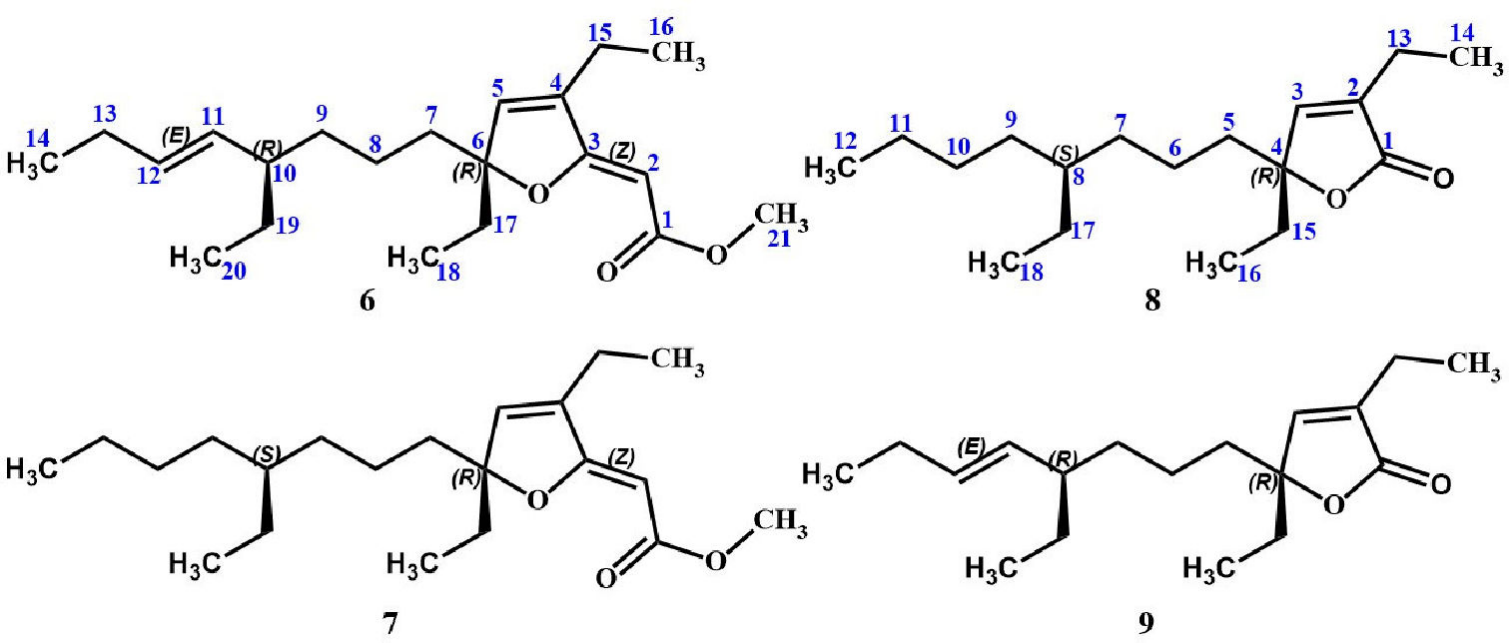
Chemical structures of the previously unknown gracilioether M (**6**), 11,12-dihydrogracilioether M (**7**), and 9,10-dihydroplakortone G (**8**), along with that of known plakortone G (**9**).

As part of our investigations, which aim to discover innovative bioactive compounds from Caribbean marine invertebrates, we recently conducted an extensive chemical analysis of the sponge *Plakortis halichondrioides*. Specimens of this species were carefully collected from waters surrounding Mona Island, which lies along the western coastline of Puerto Rico. We now wish to report the isolation and structure elucidation of three new *Plakortis* polyketides, namely, gracilioether M (**6**), 11,12-dihydrogracilioether M (**7**), and 9,10-dihydroplakortone G (**8**). Further research revealed that the latter compound was isolated together with known plakortone G (**9**) [22]. An accurate spectroscopic analysis of these compounds allowed us to establish unambiguously the specific arrangement of atoms and functional groups shown in structures **6–9** (see Figure 2).

## 2. Materials and Methods

### 2.1. Animal Material

The Caribbean marine sponge *P. halichondrioides* was collected during an underwater expedition in June 2006 near Mona Island, Puerto Rico. The specimen was kept frozen at −20 °C prior to lyophilization. A voucher specimen bearing reference number IM0619 has been meticulously preserved at the Molecular Sciences Research Center (MSRC) of the University of Puerto Rico.

### 2.2. General Experimental Procedures

Chemical reagents were procured from several suppliers, including Acros, Fluka, Sigma–Aldrich, Burlington, Massachusetts, USA, and TCI. Deuterated NMR solvents were obtained from Sigma-Aldrich. Analytical Thin-Layer Chromatography (TLC) was conducted on glass plates pre-coated with Silica Gel 60 F254 and purchased from Analtech. Visualization was achieved using UV light and/or an appropriate stain such as iodine on silica, sulfuric acid (H_2_SO_4_), or phosphomolybdic acid (PMA). Flash chromatography was carried out using Silica Gel 60 (35–75 mesh) from Analtech. Analytical reversed-phase high-performance liquid chromatography (RP-HPLC) was carried out using a HarmonySecure RP18 Agilent, Santa Clara, California, USA (250 × 4.6 mm i.d., 5 μm) column mounted to an Agilent 1260 series system controller equipped with a 1260 G1315D photodiode array detector and ChemStation software (B.04.02 SP2). Optical rotations were measured in chloroform (CHCl_3_) with an Autopol IV automatic polarimeter using a 10 mm microcell. Fourier-transform infrared (FTIR) experiments were conducted using a Bruker Tensor 27 FTIR spectrometer. Ultraviolet–visible (UV-vis) spectra were recorded using a Shimadzu UV-2401 PC UV-VIS spectrophotometer. One-dimensional (1D) and two-dimensional (2D) nuclear magnetic resonance (NMR) data were acquired in CDCl_3_ with either a Bruker DRX500 spectrometer, a Varian VS500 spectrometer, or a Varian VX500 spectrometer equipped with a Xsens Cold probe. Chemical shifts were referenced to the corresponding solvent signals (δ_H_ 7.26 and δ_C_ 77.0 for CDCl_3_). The spectra were processed using Mestrenova (Mnova 11.0 Mestrelab Research) software. All the 3D molecular modeling was performed using the Spartan 14 Parallel Suite software (version 1.1.8) running on the Microsoft Windows platform, enabling accurate visualization of all the 3D chemical structures, whereas 2D NOE correlations were incorporated using ChemDraw Prime (version 20.0.0.41).

### 2.3. Extraction and Isolation

The freeze-dried sponge was carefully cut into small chunks and blended using a mixture of chloroform (CHCl_3_) and methanol (MeOH) at room temperature. After filtration, the resulting crude extract was concentrated under reduced pressure to yield a thick brown paste. This brown material was suspended in distilled water (H_2_O) and subjected to extraction with *n*-hexane. The resulting paste was chromatographed on silica gel using mixtures of *n*-hexane and ethyl acetate (EtOAc), which led to seven fractions labeled by ascendent polarity as fractions A to G. Fraction D was tried by further chromatography separation using silica gel and a 9:1 mixture of *n*-hexane-EtOAc, yielding fractions D1 to D7. Thereafter, fraction D6 was dissolved in tetrahydrofuran (THF) and subjected to RP-HPLC using an RP18 column (5 μm, 250 × 4.6 mm i.d., 1 mL·min^−1^) and a mixture of 8:2 MeOH:H_2_O as mobile phase with the UV lamp detector set to 254 nm. Following this process, pure gracilioether M (**6**) (retention time: 19.6 min, 5.7 mg, 0.002% yield) and 11,12-dihydrogracilioether M (**7**) (retention time: 25.6 min, 3.5 mg, 0.001% yield) were isolated. The less polar fraction C was further chromatographed on silica gel using a mixture of 95:5 *n*-hexane-EtOAc to afford an inseparable mixture of 9,10-dihydroplakortone G (**8**) and previously known plakortone G (**9**) (17.5 mg, 0.006% yield).

### 2.4. Catalytic Hydrogenation of a Mixture of 9,10-Dihydroplakortone G (8)/plakortone G (9)

The mixture of 9,10-dihydroplakortone G (**8**) and plakortone G (**9**) (10.0 mg), 10% Pd-on-C (3.2 mg), and hydrogen (H_2_) gas (1 atm) in anhydrous EtOAc (3 mL) was stirred for 8 h at 25 °C. The resulting suspension was filtered through a short plug of silica gel using DCM as eluent. Following in vacuo concentration and further separation by column chromatography with silica gel and a mixture of 9:1 *n*-hexane-EtOAc, we obtained a single product, namely, 9,10-dihydroplakortone G (**8**) (0.7 mg).

### 2.5. Catalytic Hydrogenation of Gracilioether M (6)

A pure sample of gracilioether M (**6**) (1.5 mg), 10% Pd-on-C (1.5 mg), and hydrogen (H_2_) gas (1 atm) in anhydrous EtOAc (3 mL) was stirred for 8 h at 25 °C. The reaction mixture was filtered through a short plug of silica gel using DCM as eluent. After concentration in vacuo, we obtained pure 11,12-dihydrogracilioether M (**7**) (1.1 mg, 72.9% yield).

### 2.6. Pyridinium Chlorochromate (PCC) Oxidative Cleavage of Gracilioether M (6)

To a solution of gracilioether M (**6**) (1.2 mg) in 1,2-dichloroethane (10 mL), a mixture of PCC (0.03 mmol) and 4 Å molecular sieves (10 mg) was added. The resulting mixture was heated to reflux and allowed to react for a period of 15 h following a known procedure [23]. Thereafter, the reaction mixture was filtered through a short plug of silica gel using DCM as eluent. The subsequent concentration of the eluate yielded pure plakortone G (**9**) (0.6 mg, 60.1% yield).

### 2.7. Pyridinium Chlorochromate (PCC) Oxidative Cleavage of 11,12-Dihydrogracilioether M (7)

To a solution of 11,12-dihydrogracilioether M (**7**) (1.3 mg) in 1,2-dichloroethane (10 mL), a mixture of PCC (0.03 mmol) and 4 Å molecular sieves (10 mg) was added. The reaction mixture was heated to reflux and allowed to react for a period of 15 h following a known procedure [23]. Thereafter, the reaction mixture was filtered through a short plug of silica gel using DCM as eluent. The subsequent concentration of the eluate yielded pure 9,10-dihydroplakortone G (**8**) (0.7 mg, 64.8% yield).

### 2.8. Antiplasmodial Activity against the Parasite Plasmodium Berghei

The antiplasmodial activity of gracilioether M (**6**), 11,12-dihydrogracilioether M (**7**), and the mixture of 9,10-dihydroplakortone G (**8**) and plakortone G (**9**) was examined using the *P. berghei* GFP-Lucama1 (1037cl1) parasite line, achieving the half-maximal effective concentration (EC_50_) by an in vitro drug luminescence assay [24,25]. This experiment was standardized utilizing the chloroquine diphosphate salt (100 nM, Sigma-Aldrich) as the control group to determine the inhibition of blood stage development. The stock solution of the specific glutathione S-transferase (GST) inhibitors (S-hexyl glutathione and ellagic acid, Sigma-Aldrich) was prepared in 100% dimethyl sulfoxide (DMSO). Compounds **6** and **7,** as well as the inseparable mixture of **8** and **9,** were individually dissolved in 100% DMSO, leading to stock solutions in a concentration of 10 mM for each compound. These stock solutions were aliquoted and stored at −20 °C before the experiments. Additionally, further dilutions of our three samples from the stock solutions were prepared using the Roswell Park Memorial Institute medium (RPMI 1640) supplemented with 20% heat-inactivated fetal bovine serum (FBS, Gibco, Grand Island, New York, USA) and 10,000 IU/mL of neomycin solution (Sigma-Aldrich). Each natural product solution was prepared 24 h before the experiment in a concentration of 10 μM and stored at 4 °C. Experiments were conducted in triplicate. Furthermore, the experiments and the data analysis were performed as described in previous works using GraphPad Prism vs. 6 software [24,25].

### 2.9. In Vitro Cell Viability Assay

Cell culture method: The human breast cancer cell line MCF-7 from ATCC^®^ was used to perform the cell viability assay. The cells were stored under liquid nitrogen at the MSRC until experiments were conducted. For the experiments, MCF-7 cells were thawed and grown in Dulbecco’s Modified Eagle’s Medium (DMEM) employing 5 % FBS and 1% penicillin-streptomycin (Pen-Strep) and incubated at 37 °C in 5% carbon dioxide (CO_2_). Cell passages were conducted twice weekly every 7 days. The cells were left to rise by 80% of confluency and appropriated to perform the cell viability experiments. This cell culture method was adapted from the ATCC product information and other previous works [26,27].

Cell viability test: Two-fold serial dilutions of compounds gracilioether M (**6**), 11,12-dihydrogracilioether M (**7**), and the mixture of 9,10-dihydroplakortone G (**8**) and plakortone G (**9**) in a concentration range of 0–400 μM were previously prepared to determine the IC_50_. Subsequently, MCF-7 cells were seeded in 96-well plates at a 5.0 × 103 cells/mL density. Cells were incubated for 24 h at 37 °C in 5% CO_2_, allowing for cell adherence to the 96-well plate. After 24 h of seeding, MCF-7 cells were treated with 100 μL of compounds **6** and **7** and the mixture of compounds **8** and **9** (0–400 μM). The control group was treated with just media (DMEM, 1% Pen-Strep). The cell viability was determined after 72 h of treatment. AlamarBlue^®^ assay was employed to determine the cell viability; the media was exchanged with 100 μL of AlamarBlue^®^ solution (10%). After exchanging the media, the 96-well plates were incubated for 4 h. The fluorescence (TECAN Microplate Reader Infinite M200) was measured at 560.0 nm of excitation and λ_max_ = 590.0 nm of emission. The MCF-7 metabolic active cells (cells under proliferation) were analyzed, taking into consideration the viability of the control group (100%) compared with the MCF-7 cells treated with compounds **6** and **7**, as well as the mixture of compounds **8** and **9**. The IC_50_ was determined for these compounds by generating dose–response curves (% live cells vs. concentration), employing GraphPad Prism vs. 9.3.0 and a nonlinear regression method. The experiments were performed in triplicate, and the mean, standard deviation, and coefficient of variation (%CV) were reported. This cell viability assay was adapted from previous studies [26,27].

## 3. Results

### 3.1. Chemical Structural Analysis

During this study, three new polyketides possessing a furanylidene motif were isolated from the marine sponge *P. halichondrioides* along with previously known plakortone G (**9**) [22], thus totaling four compounds. Gracilioether M (**6**) was obtained as a colorless optically active oil: [α]^20^_D_-80.5 (c 0.96, CHCl_3_); UV (MeOH) λ_max_ (log ε) 285 (3.45), 240 (2.55), 201 (3.17) nm; and _max_ (thin film) 2964, 2936, 2874, 1715, 1689, 1626, 1459, 1164 cm^−1^. High-resolution electrospray ionization mass spectrometry (HRESIMS) indicated a single [M+Na]^+^ ion peak at *m/z* 357.2401, suggesting a molecular formula of C_21_H_34_O_3_ (calculated as 357.2400). Likewise, 11,12-dihydrogracilioether M (**7**) was obtained as a colorless optically active oil: [α]^20^_D_-47.1 (c 0.70, CHCl_3_); UV (MeOH) λ_max_ (log ε) 286 (3.15), 240 (2.31) nm; and _max_ (thin film) 2961, 2929, 2873, 2859, 1716, 1688, 1638, 1626, 1458, 1273, 1164 cm^−1^. HRESIMS indicated a single [M+Na]^+^ ion peak at *m/z* 359.2553, suggesting a molecular formula of C_21_H_36_O_3_ (calcd 359.2557). All the proton and carbon resonances of **6** and 7 were assigned by detailed 1D ^1^H- and ^13^C-NMR analysis and a combination of 2D NMR [^1^H–^1^H-correlated spectroscopy (^1^H–^1^H COSY), heteronuclear single-quantum coherence spectroscopy (HSQC), and heteronuclear multiple-bond correlation (HMBC)]. The ^1^H and ^13^C NMR data for compounds **6** and **7** are shown in Table 1.

The two-dimensional molecular structures for gracilioether M (**6**) and 11,12-dihydrogracilioether M (**7**) were successfully determined based on gradient-selected correlation spectroscopy (gCOSY), total correlation spectroscopy (TOCSY), HSQC, and HMBC data. Copies of all the spectra can be found in the supplementary materials as Figures S1–S14. Careful spectroscopic analyses clearly show a distinction between the two compounds: gracilioether M (**6**) possessed an additional olefin at the C11–C12 position (δ_H_ 5.02, H11; δ_H_ 5.34, H12; δ_C_ 133.3, C11; δ_C_ 132.2, C12) when compared against 11,12-dihydrogracilioether M (**7**) (δ_H_ 1.10–1.20, H11α; δ_H_ 1.10–1.20, H11β; δ_H_ 1.10–1.20, H12; δ_C_ 32.7, C11; δ_C_ 28.8, C12). However, it is important to mention that the furanylidene functionality present in **6** and **7** is the same as that reported for compounds **1–5** [17–20] on account of the fact that all these compounds display the same key resonances, which are ascribable to that functionality.

Conversely, despite our best efforts, 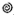,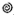-unsaturated butyrolactones 9,10-dihydroplakortone G (**8**) and plakortone G (**9**) were isolated as an inseparable mixture. This mixture was obtained as a yellowish optically active oil: [α]^20^_D_-38.3 (c 0.87, CHCl_3_). The evaluation of the HRESIMS of the mixture suggested that it consisted of two compounds with formulae C_18_H_30_O_2_ ([M+Na]^+^ *m/z* 301.2138 found, 301.2138 calcd) and C_18_H_32_O_2_ ([M+Na]^+^ *m/z* 303.2299 found, 303.2295 calcd) for **8** and **9**, respectively. The evaluation of the NMR data for compound **9** (shown in Table S1 in the supplementary materials) indicated that it matched those reported for plakortone G [22]. Likewise, the structure of compound **8** was demonstrated by a combination of 2D NMR (^1^H–^1^H COSY, HSQC, HMBC) and high-resolution mass spectrometry data. The ^1^H and ^13^C NMR data for compound **8** are reported in Table 1.

### 3.2. In Vitro Drug luminescence Assay against Plasmodium Berghei

**Figure 3.**
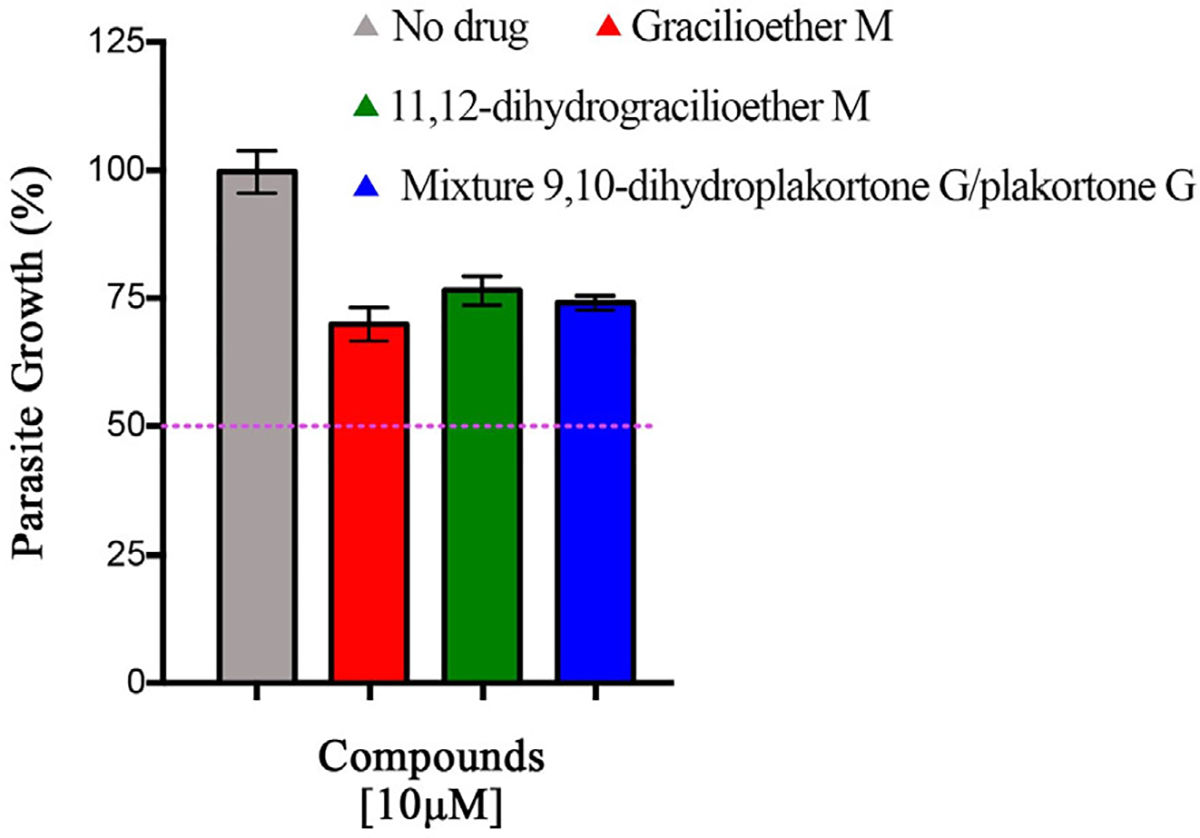
Results for antiplasmodial activity at 10 μM. These data represent one experiment in triplicate. Bars represent the standard deviation. None of the compounds tested inhibited parasite growth by 50% at 10 μM.

The evaluation of gracilioether M (**6**), 11,12-dihydrogracilioether M (**7**), and the mixture of 9,10-dihydroplakortone G (**8**) and plakortone G (**9**) for antiplasmodial activity was assessed through an in vitro drug luminescence assay against *P. berghei* [24,25]. The *P. berghei* model is typically used to initially test potential antiplasmodial compounds in research and is one of the preclinical steps in drug development [28–30]. *P. berghei* is closely related to human malaria parasites, as their life cycles and pathophysiology are similar [31]. Additionally, the complete life cycle is easy to maintain in laboratory settings [32]. Figure 3 illustrates the results obtained for *Plakortis* metabolites **6–9** at 10 μM. It was observed that the percentage of parasite growth decreased to ~70–75 % when compared with the control group (100%). However, these findings revealed that none of the compounds tested inhibited parasite growth by >50% at 10 μM, which indicated that these marine natural products did not possess significant antiplasmodial activity when compared against chloroquine (positive control, EC_50_ = 23.23 nM, the dose–response curve for chloroquine is included as Figure S23 in the supplementary materials), a common drug employed for the treatment of malaria [33]. Due to the lack of significant antiplasmodial activity of compounds **6–9**, the EC_50_ values of these compounds were not determined. However, the dose–response curves are included as Figures S24–S26 (see Supplementary Materials).

### 3.3. In Vitro Cytotoxicity Assay against MCF-7 Cell Line

The in vitro cytotoxicity assessment for gracilioether M (**6**), 11,12-dihydrogracilioether M (**7**), and the mixture of 9,10-dihydroplakortone G (**8**) and plakortone G (**9**) was conducted employing a human breast cancer MCF-7 cell line after 72 h of treatment. These experiments were performed using a concentration range of 0–400 μM. Table 2 provides a summary of the percentages of cell viability and the coefficients of variation (%CV) resulting from the MCF-7 cells treated with compounds **6** and **7** and the mixture of compounds **8** and **9**.

**Figure 4.**
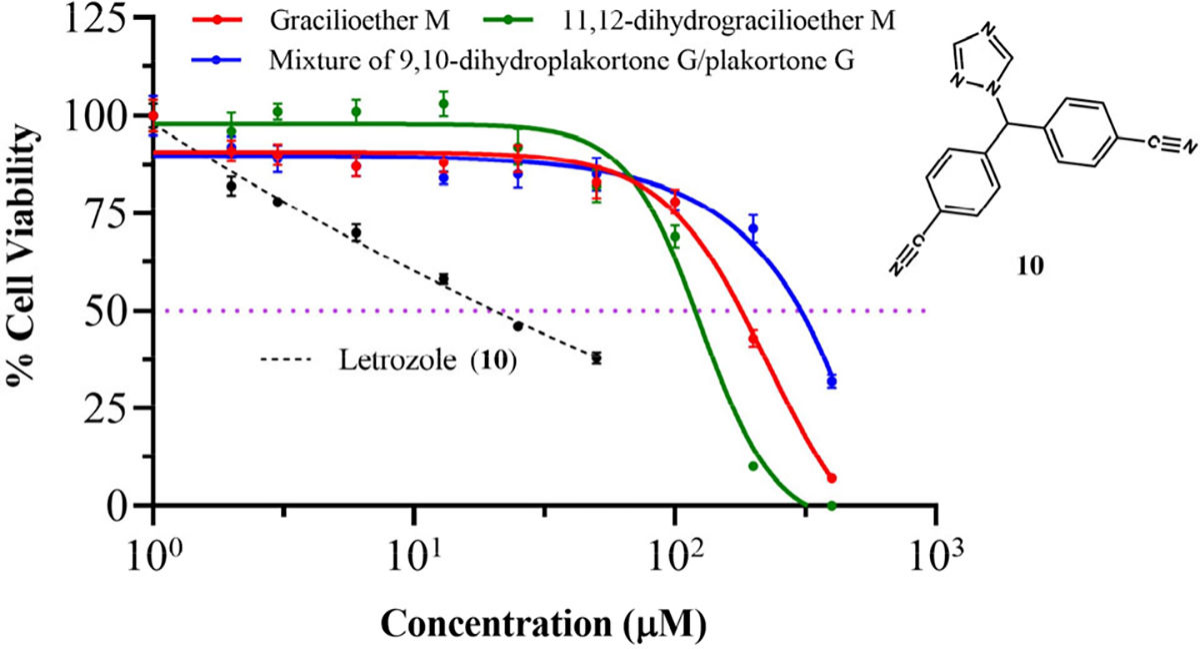
Dose–response or IC_50_ curves of the human breast cancer MCF-7 cell line for (red) compound **6** (gracilioether M), (green) compound **7** (11,12-dihydrogracilioether M), (blue) the mixture of compound **8** and **9** (9,10-dihydroplakortone G and plakortone G), and (black dashed lines) letrozole (**10**) as positive control.

Figure 4 illustrates the dose–response curve (IC_50_ curves) of MCF-7 cells after 72 h of treatment. The cell viability assays reveal IC_50_ values > 100 μM for all the analyzed marine natural products after treating the human breast cancer MCF-7 cells. The measured IC_50_ values were 169, 119, and 299 μM for gracilioether M (**6**), 11,12-dihydrogracilioether M (**7**), and the mixture of 9,10-dihydroplakortone G (**8**) and plakortone G (**9**), respectively. These findings demonstrate that the title compounds do not display significant cytotoxicity effects against MCF-7 cells when compared with letrozole (**10**) (positive control, IC_50_ = 20 μM) [27], a drug that is usually prescribed for the treatment of estrogen receptor-positive (ER-positive) breast cancer subtypes, represented here by MCF-7 cells [34].

## 4. Discussion

**Figure 5.**
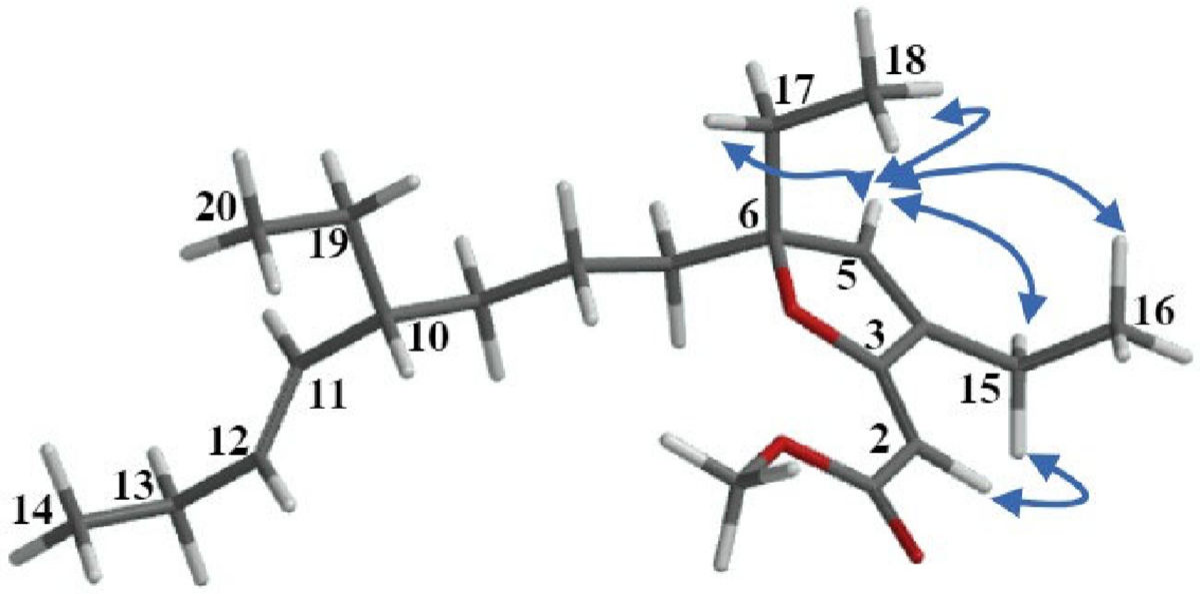
Selected NOE interactions observed in the 2D NOESY spectra for gracilioether M (**6**).

Once the planar molecular structures of the designated natural products were elucidated as depicted in **6–8** based on the analyses outlined above, our attention was diverted toward establishing their relative stereochemistry. Two out of the twenty-one carbons of **6** and **7** are stereogenic, and their location along the C8 acyclic tail made the configurational assignment somewhat difficult. Initially, the relative configurations of the stereocenters at C6 and C10 in compounds **6** and 7 were tentatively established through comprehensive analyses that included comparisons of proton and carbon chemical shifts along with proton NMR coupling data between each of these compounds (Table 1) and those reported for compound **1** [17]. Furthermore, a 2D NOESY experiment allowed us to reduce the number of possible stereoisomers surrounding the furanylidene moiety. Figure 5 highlights a few of the 2D NOESY cross-peaks that were observed for compound **6**. The actual 2D NOESY NMR spectrum of **6** (Figure S8) has been included in the Supplementary Materials. Most notably, the presence of cross-peaks between H_2_-15 and both H2 and H5 indicated that these protons lie within close proximity to each other in both gracilioether M (**6**) and 11,12-dihydrogracilioether M (**7**).

Regarding the C6 and C10 in compounds **6** and **7**, the absence of reliable cross-peaks that were ascribable to Hs near these centers made it impossible to assign their relative stereochemistry based on NOE correlations alone. Notwithstanding this, the fact that we managed to convert **6** into **7** by selective catalytic hydrogenation (vide infra) proved categorically that these compounds share a common stereochemistry at C6 and C10. At this time, we remark that this conclusion was later substantiated through detailed comparisons of relevant NMR chemical shifts of compound **7** with those of 9,10-dihydroplakortone G (**8**) (see Table 1).

**Scheme 1.**
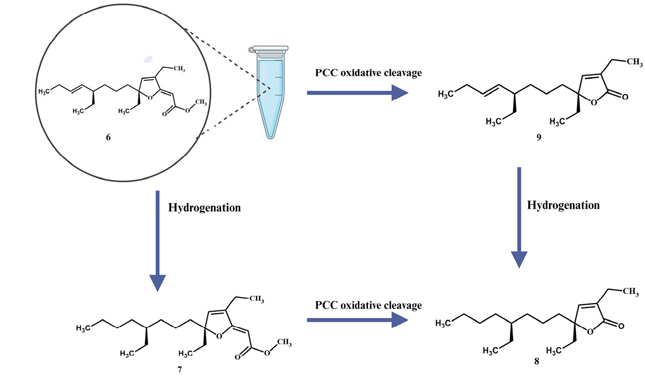
11,12-dihydrogracilioether M (**7**), 9,10-dihydroplakortone G (**8**), and plakortone G (**9**). Illustration created with Biorender.com.

To finalize our configurational assignments for compounds **6** and **7,** we conducted a series of chemical correlation studies (Scheme 1) inspired by the prior work of Kowashi and coworkers [35], who had firmly established the absolute configuration of plakortone G (**9**) as 4*R*,8*R*. We commenced our investigation by conducting the catalytic hydrogenation of the mixture of 9,10-dihydroplakortone G (**8**) and plakortone G (**9**) (H_2_, Pd-on-C, EtOAc, 8 h). This simple procedure led to compound **8** as the sole product and thus quickly established its absolute configuration as 4*S*,8*R*. Thereafter, the absolute configuration for gracilioether M (**6**) and 11,12-dihydrogracilioether M (**7**) at C6 and C10 could be inferred following a similar strategy. In the first place, we successfully transformed compounds **6** and **7** into plakortone G (**9**) and 9,10-dihydroplakortone G (**8**), respectively, through chemoselective PCC oxidative cleavage, as outlined by Perkins [23] [PCC (0.03 mmol), 4 Å MS (10.0 mg), 1,2-dichloroethane (10 mL), reflux 15 h]. Since we had already succeeded at converting **6** into **7** by selective catalytic hydrogenation, the **6 9** and **7 8** degradations effectively established the absolute stereochemistry of all the new compounds. A summary of all the chemical interconversions conducted during this investigation is shown in Scheme 1. In the second place, in so far as the configurational assignments for the ^2^ and ^11^ olefins in **6** (or ^2^ in **7**) are concerned, we used the following strategy: The (*Z*)-geometry shown at ^2^ in **6** and **7** was based on the presence of strong nuclear Over-hauser effect (NOE) interactions between the protons attached to C2 and C15 (Figure 5). Regarding the geometry of ^11^ in **6**, the large coupling constant (15.1 Hz) detected between protons H11 and H12 combined with the conspicuous absence of NOEs between them support the (*E*)-geometry.

Arguably, the lack of activity of **6–9** against *P. berghei* might be attributed to the absence of an endoperoxide ring system in them. Prior research has consistently highlighted the pivotal role of this specific structural feature in conferring antimalarial activity [36,37]. The endoperoxide ring system is known for its ability to generate reactive oxygen species within the parasiteʹs cells, ultimately leading to the destruction of the malaria-causing pathogen [38–41]. Hence, the absence of this moiety in compounds **6** and **7** and the mixture of **8** and **9** aligns favorably with the observed lack of activity against the malaria parasite. This finding underscores the importance of the endoperoxide motif in the design and development of antimalarial agents and provides valuable insights for future research in this field.

As far as our in vitro cell viability experiments are concerned, we noticed that our data revealed that none of the tested natural marine products exhibited discernible anticancer activity against the cell line used throughout this work. The reduced effectiveness of compounds **6–9** against MCF-7 cells may be explained by the absence of hydrophilicity in their molecular structures. This contrasts with letrozole (**10**), which contains several nitrile and 1H-1,2,4-triazole groups, as indicated in Figure 4. These functional groups within structure **10** provide multiple sites for potential interactions through hydrogen bonding within the enzyme pocket site of MCF-7 cells [42].

**Figure 6.**
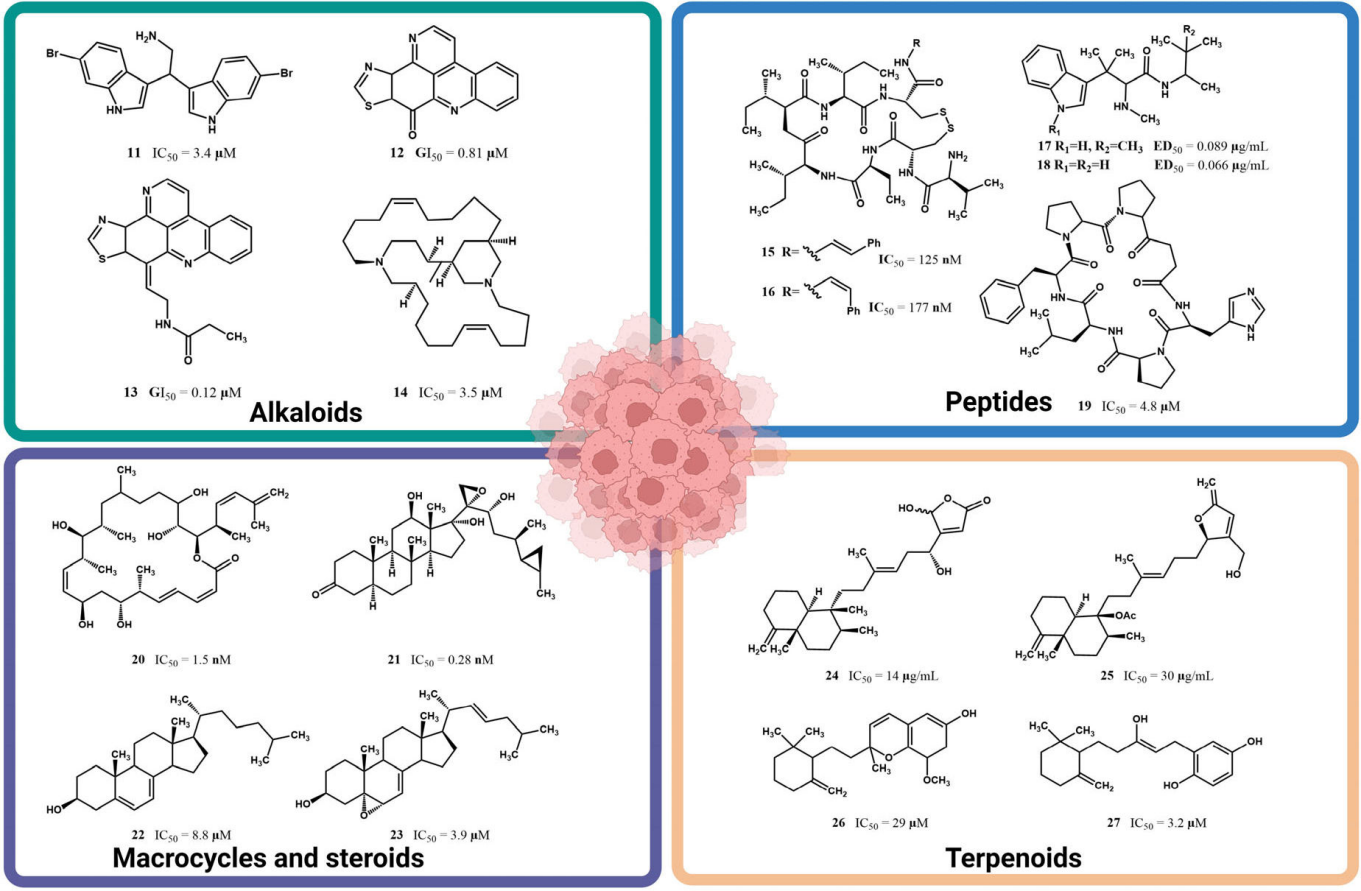
Natural marine products with significant activity against human breast cancer MCF-7 cell lines. The compilation shown was achieved using partial data provided by Hussain et al. [45] and corroborated at the sources. Illustration created with Biorender.com.

The observation regarding a plausible hydrophilic binding in the enzyme pocket site of MCF-**7** cells finds support in the fact that several natural marine products, renowned for their remarkable anticancer activity against this breast cancer cell line, exhibit even lower IC_50_ values than those reported for letrozole (**10**), since they also feature highly hydrophilic functional groups throughout their molecular structures. For instance, 2,2-bis(6′-bromo-3′-indolyl)ethylamine (**11**), an alkaloid that was isolated for the first time from the marine tunicate *Didemnum candidum* [43] and later reisolated from the marine sponge *Gellius sp*., demonstrated an IC_50_ of 3.4 μM against MCF-7 cells [44]. The natural marine products depicted in Figure 6, which include alkaloids such as 2,2-bis(6′-bromo-3′-indolyl)ethylamine (**11**), kuanoniamine A (**12**), kuanoniamine C (**13**), and neopetrosiamine A (**14**), peptides like microcianamide A (**15**), microciamide B (**16**), hemiasterlin A (**17**), hemiasterlin B (**18**), and stylissatin B (**19**), the macrocycle polyketide disctyostatin-1 (**20**), steroids such as aragusterol A (**21**) and sterols **22–23**, and terpenoids like palaluolol (**24**), thorectandrol B (**25**), metachromin U (**26**), and metachromin (**27**), were sourced from the research conducted by Hussain et al. [45]. These compounds have been spotlighted for their substantial activity against MCF-7 cells. They all share highly hydrophilic functional groups throughout their molecular structures, thus facilitating strong interactions through hydrogen bonding. In contrast, compounds **6–9** lack such an affinity due to their extensive hydrophobic carbon chain, making these chemical interactions difficult. Figure 6 provides an overview of the chemical structures of some representative natural products that were isolated from marine sponges with significant activities against MCF-7 cells. The corresponding IC_50_, IG_50_, or ED_50_ values for these compounds are also showcased in Figure 6.

Equally important, the human breast cancer MCF-7 cells employed during this work represent one of the most common types of breast cancer as an ER-positive breast cancer subtype that expresses estrogen receptors [34,46]. Letrozole, employed here as the control positive, is a type II aromatase inhibitor that prevents the transformation of androgens to estrogens (required for breast cancer cell proliferation) using the aromatase enzyme [47,48]. It is conceivable that the absence of cytotoxic activity in compounds **6–9** could be explained due to their marked differences in molecular structure when contrasted with letrozole, thus causing them to be ineffective inhibitors of the aromatase enzyme.

## 5. Conclusions

Two new polyketides containing the [2(5H)-furanylidene]ethanoate moiety, gracilioether M (**6**) and 11,12-dihydrogracilioether M (**7**), along with new 9,10-dihydroplakortone G (**8**), were isolated from the *n*-hexane extract of the Caribbean sponge *P. halichondrioides*. Their molecular structures, including absolute configuration, were characterized using a combination of spectroscopic analysis and chemical correlation studies that involved well-known reactions such as PCC oxidative cleavage and catalytic hydrogenation. These methods also allowed us to assign the correct geometry for the ^2^ and ^11^ stereocenters in **6** and **7**. The lead molecules identified herein belong to a large class of bioactive polyketide natural products that become highly diversified through the formation and decomposition of cyclic endoperoxide intermediates [11,49]. To date, members of this family of natural products include spongosoritin A [17,18] and the related plakilactones or gracilioethers [19,50]. Herein, we report that compounds **6** and **7**, and a mixture of compounds **8** and **9,** failed to inhibit 50% of *P. berghei*’s growth at a concentration of 10 μM. The lack of a biological response is likely due to the absence of an endoperoxide moiety in the molecular structure of these compounds. Additional experiments are required to fully understand the absence of cytotoxicity in gracilioether M (**6**), 11,12-dihydrogracilioether M (**7**), and 9,10-dihydroplakortone G (**8**) against human breast cancer cells. Unfortunately, the scarcity of the new compounds isolated hindered our efforts to further investigate the potential impact of these compounds on other human breast cancer cell lines. This predicament severely limits any future perspectives.

## Supplementary Material

Supp

## Figures and Tables

**Table 1.** NMR spectroscopic data for gracilioether M (6), 11,12-dihydrogracilioether M (7), and 9,10-dihydroplakortone G (8) in CDCl_3_ *.

Atom	Compound 6		Compound 7		Compound 8	

	δC ^a^	δH ^b^ (*J* in Hz)	δC ^a^	δH ^b^ (*J* in Hz)	δC ^a^	δH ^b^ (*J* in Hz)

1	166.9, C		166.9, C		173.5, C	
2	83.4, CH	4.81, s	83.5, CH	4.82, s	135.8, C	
3	171.9, C		171.9, C		150.1, CH	6.83, ovl^c^
4	139.9, C		140.0, C		89.3, C	
5	139.7, CH	6.22, s	139.7, CH	6.23, s	36.9, CH_2_	1.73, m
						1.73, m
6	98.0, C		97.9, C		20.6, CH_2_	1.10–1.38, ovl ^c^
7	38.1, CH_2_	1.65, m	37.8, CH_2_	1.65, m	33.3, CH_2_	1.38, ovl ^c^
		1.78, m		1.78, m		1.38, ovl ^c^
8	21.4, CH_2_	1.10–1.30, ovl c	20.9, CH_2_	1.10–1.30, ovl c	38.6, CH	1.10–1.38, ovl ^c^
9	35.1, CH_2_	1.29, m	33.1, CH_2_	1.30, ovl ^c^	33.2, CH_2_	1.38, ovl ^c^
		1.29, m		1.30, ovl ^c^		1.38, ovl ^c^
10	44.3, CH	1.72, m	38.6, CH	1.10–1.30, ovl ^c^	28.8, CH_2_	1.10–1.38, ovl ^c^
11	133.2, CH	5.01, dd (8.8, 15.2)	32.7, CH_2_	1.30, ovl ^c^	23.0, CH_2_	1.10–1.38, ovl^c^
				1.30, ovl ^c^		
12	132.1, CH	5.33, m	28.8, CH_2_	1.10–1.30, ovl ^c^	14.1, CH_3_	0.86, t (7.4)
13	25.6, CH_2_	1.97, m	23.0, CH_2_	1.10–1.30, ovl ^c^	18.5, CH_2_	2.28, m
14	14.2, CH_3_	0.94, t (7.5)	14.1, CH_3_	0.87, t (7.2)	12.0, CH_3_	1.15, t (7.5)
15	18.5, CH_2_	2.17, m	18.5, CH_2_	2.17, m	29.9, CH_2_	1.70, m
						1.79, m
16	12.0, CH_3_	1.15, t (7.5)	12.0, CH_3_	1.15, t (7.5)	7.7, CH_3_	0.80, m
17	30.8, CH_2_	1.72, m	30.8, CH_2_	1.75, m	30.0, CH_2_	1.38, ovl ^c^
		1.84, m		1.84, m		1.38, br m
18	8.0, CH_3_	0.79, m	8.0, CH_3_	0.79, m	10.7, CH_3_	0.80, m
19	28.1, CH_2_	1.30, ovl ^c^	30.9, CH_2_	1.30, ovl ^c^		
		1.30, ovl ^c^		1.30, ovl ^c^		
20	11.6, CH_3_	0.79, m	10.7, CH_3_	0.79, m		
21	50.5, CH_3_	3.68, s	50.5, CH_3_	3.68, s		

* All assignments are based on COSY, HSQC, and HMBC experiments.

a Recorded at 125 MHz. Multiplicities were obtained from the Attached Proton Test (APT) experiments.

b Recorded at 500 MHz.

c Overlapped signal.

**Table 2.** Percentages of cell viability of MCF-7 cells treated with compound **6** (gracilioether M), compound **7** (11,12-dihydrogracilioether M), and the mixture of compounds **8** (9,10-dihydroplakortone G) and **9** (plakortone G).

Concentration (μM)	Compound 6		Compound 7		Mixture of Compounds 8 and 9	

	% Cell Viability	% CV	% Cell Viability	% CV	% Cell Viability	% CV

400	7	5	0	0	32	5
200	43	5	10	3	71	5
100	78	4	69	4	78	3
50	83	5	82	5	85	5
25	89	4	92	5	85	4
13	88	3	103	3	84	2
6	87	3	101	3	87	3
3	90	3	101	2	89	4
2	91	3	96	5	92	3
0	100	4	100	4	100	5

## Data Availability

The data presented in this study are available in this article.

## References

[1] Newman DJ; Cragg GM Marine Natural Products and Related Compounds in Clinical and Advanced Preclinical Trials. J. Nat. Prod. 2004, 67, 1216–1238. 15332835 10.1021/np040031y

[2] Altmann KH Drugs from the Oceans: Marine Natural Products as Leads for Drug Discovery. Chimia 2017, 71, 646–651. 29070409 10.2533/chimia.2017.646

[3] Montaser R; Luesch H Marine Natural Products: A New Wave of Drugs? Future Med. Chem 2011, 3, 1475–1489. 21882941 10.4155/fmc.11.118 PMC3210699

[4] Mayer AMS; Pierce ML; Howe K; Rodríguez AD; Taglialatela-Scafati O; Nakamura F; Fusetani N Marine Pharmacology in 2018: Marine Compounds with Antibacterial, Antidiabetic, Antifungal, Anti-Inflammatory, Antiprotozoal, Antituberculosis and Antiviral Activities; Affecting the Immune and Nervous Systems, and Other Miscellaneous Mechanisms of Action. Pharmacol. Res. 2022, 183, 106391. 35944805 10.1016/j.phrs.2022.106391

[5] Newman DJ; Cragg GM Natural Products as Sources of New Drugs over the Nearly Four Decades from 01/1981 to 09/2019. J. Nat. Prod. 2020, 83, 770–803. 32162523 10.1021/acs.jnatprod.9b01285

[6] Lewis K Platforms for Antibiotic Discovery. Nat. Rev. Drug Discov. 2013, 12, 371–387. 23629505 10.1038/nrd3975

[7] Wright AD; König GM; Angerhofer CK; Greenidge P; Linden A; Desqueyroux-Faúndez R Antimalarial Activity: The Search for Marine-Derived Natural Products with Selective Antimalarial Activity. J. Nat. Prod. 1996, 59, 710–716. 8759172 10.1021/np9602325

[8] Fattorusso E; Taglialatela-Scafati O Marine Antimalarials. Mar. Drugs 2009, 7, 130–152. 19597577 10.3390/md7020130 PMC2707039

[9] Karns AS; Ellis BD; Roosen PC; Chahine Z; Le Roch KG; Vanderwal CD Concise Synthesis of the Antiplasmodial Isocyanoterpene 7,20-Diisocyanoadociane. Angew. Chem. Int. Ed. 2019, 58, 13749–13752. 10.1002/anie.201906834 PMC6759395 31270921

[10] Rahm F; Hayes PY; Kitching W Metabolites from Marine Sponges of the Genus Plakortis. Heterocycles 2004, 64, 523–575.

[11] Chianese G; Yu HB; Yang F; Sirignano C; Luciano P; Han BN; Khan S; Lin HW; Taglialatela-Scafati O PPAR Modulating Polyketides from a Chinese *Plakortis simplex* and Clues on the Origin of Their Chemodiversity. J. Org. Chem. 2016, 81, 5135–5143. 27232542 10.1021/acs.joc.6b00695

[12] Batista ANL; Dos Santos FM; Valverde AL; Batista JM Stereochemistry of Spongosoritins: Beyond Optical Rotation. Org. Biomol. Chem. 2019, 17, 9772–9777. 31710062 10.1039/c9ob02010a

[13] Kossuga MH; Nascimento AM; Reimão JQ; Tempone AG; Taniwaki NN; Veloso K; Ferreira AG; Cavalcanti BC; Pessoa C; Moraes MO; Antiparasitic, Antineuroinflammatory, and Cytotoxic Polyketides from the Marine Sponge *Plakortis angulospiculatus* Collected in Brazil. J. Nat. Prod. 2008, 71, 334–339. 18177008 10.1021/np0705256

[14] Jiménez-Romero C; Rodríguez AD; Nam S Plakortinic Acids A and B: Cytotoxic Cycloperoxides with a Bicyclo[4.2.0]Octene Unit from Sponges of the Genera *Plakortis* and *Xestospongia*. Org. Lett. 2017, 19, 1486–1489. 28272898 10.1021/acs.orglett.7b00547 PMC9126020

[15] Jiménez-Romero C; Amador LA; Rodríguez AD Plakortinic Acids C and D: A Pair of Peroxide-Polyketides Possessing a Rare 7,8-Dioxatricyclo[4.2.2.02,5]Dec-9-Ene Core from a Two-Sponge Association of *Plakortis symbiotica*–*Xestospongia deweerdtae*. Tetrahedron Lett. 2021, 66, 3–6. 10.1016/j.tetlet.2021.152833 PMC7935052 33678913

[16] Wei X; Ding Y; An F A New Polyketide from Marine-Derived *Paraconiothyrium* sp. *Nat. Prod. Commun*. 2022, 17, 1934578X221075986.

[17] Capon RJ; Singh S; Ali S; Sotheeswarun S Spongosoritin A: A New Polyketide from a Fijian Marine Sponge, *Spongosorites* sp. Aust. J. Chem. 2005, 58, 18–20.

[18] Epifanio RDA; Pinheiro LS; Alves NC Polyketides from the Marine Sponge Plakortis angulospiculatus. J. Braz. Chem. Soc 2005, 16, 1367–1371.

[19] Ueoka R; Nakao Y; Kawatsu S; Yaegashi J; Matsumoto Y; Matsunaga S; Furihata K; Van Soest RWM; Fusetani N Gracilioethers A-C, Antimalarial Metabolites from the Marine Sponge *Agelas gracilis*. J. Org. Chem. 2009, 74, 4203–4207. 19402618 10.1021/jo900380f

[20] Duggan BM; Cullum R; Fenical W; Amador LA; Rodríguez AD; La Clair JJ Searching for Small Molecules with an Atomic Sort. Angew. Chem. Int. Ed. 2020, 59, 1144–1148. 10.1002/anie.201911862 PMC6942196 31696595

[21] Norris MD; Perkins MV A Biomimetic Cascade for the Formation of the Methyl [2(5H)-Furanylidene] Ethanoate Core of Spongosoritin A and the Gracilioethers. Tetrahedron 2013, 69, 9813–9818.

[22] Gochfeld DJ; Hamann MT Isolation and Biological Evaluation of Filiformin, Plakortide F, and Plakortone G from the Caribbean Sponge *Plakortis* sp. J. Nat. Prod. 2001, 64, 1477–1479. 11720540 10.1021/np010216u

[23] Norris MD; Perkins MV Total Synthesis of Plakilactones C, B and Des-Hydroxyplakilactone B by the Oxidative Cleavage of Gracilioether Furanylidenes. J. Org. Chem. 2016, 81, 6848–6854. 27359169 10.1021/acs.joc.6b01196

[24] Lin J; Sajid M; Ramesar J; Khan SM; Janse CJ; Franke-Fayard B Screening Inhibitors of *P. berghei* Blood Stages Using Bioluminescent Reporter Parasites. Methods Mol. Biol. 2012, 923, 507–522. 10.1007/978-1-62703-026-7_35 22990801

[25] Colón-Lorenzo EE; Colón-López DD; Vega-Rodríguez J; Dupin A; Fidock DA; Baerga-Ortiz A; Ortiz JG; Bosch J; Serrano AE Structure-Based Screening of Plasmodium Berghei Glutathione S-Transferase Identifies CB-27 as a Novel Antiplasmodial Compound. Front. Pharmacol. 2020, 11, 246. 32256353 10.3389/fphar.2020.00246 PMC7090221

[26] Carmona-Sarabia L; Quiñones Vélez G; Escalera-Joy AM; Mojica-Vázquez D; Esteves-Vega S; Peterson-Peguero EA; López-Mejías V Design of Extended Bisphosphonate-Based Coordination Polymers as Bone-Targeted Drug Delivery Systems for Breast Cancer-Induced Osteolytic Metastasis and Other Bone Therapies. Inorg. Chem. 2023, 62, 9440–9453. 37278598 10.1021/acs.inorgchem.3c00542

[27] Carmona-sarabia L; Quiñones Vélez G; Mojica-Vázquez D; Escalera-Joy A; Esteves-Vega S; Peterson EA; Lopez-Mejias V High-Affinity Extended Bisphosphonate-Based Coordination Polymers as Promising Candidates for Bone-Targeted Drug Delivery. ACS Appl. Mater. Interfaces 2023, 15, 33397–33412. 37404172 10.1021/acsami.3c05421

[28] Raether W; Enders B; Hofmann J; Schwannecke U; Seidenath H; Hänel H; Uphoff M Antimalarial Activity of New Floxacrine-related Acridinedione derivatives: Studies on Blood Schizontocidal Action of Potenctial Candidates Against *P. berghei* in Mice and *P. falciparum* in vivo and in vitro. Parasitol. Res. 1989, 75, 619–626. 2671988 10.1007/BF00930959

[29] Smith PW; Diagana TT; Yeung BKS Progressing the Global Antimalarial portfolio: Finding Drugs with Target Multiple *Plasmodium* Life Stages. Parasitology 2014, 141, 66–76. 23746048 10.1017/S0031182013000747

[30] Calit J; Araújo JE; Deng B; Miura K; Gaitán X; da Silva Aráujo M; Medeiros JF; Long CA; Simeonov A; Eastman RT; Novel Transmission-Blocking Antimalarials Identified by High-Throughput Screening of *Plasmodium berghei* Oocluc. Antimicrob. Agents Chemother. 2023, 67, e014655–22. 10.1128/aac.01465-22 PMC10112123 36856421

[31] Rathnapala UL; Goodman CD; McFadden GI A Novel Genetic Technique in *Plasmodium berghei* Allows Liver Stage Analysis of Genes Required for Mosquito Stage Development and Demonstrates that the novo Heme Synthesis is Essential for Liver Stage Development in the Malaria Parasite. PLoS Pathog. 2017, 13, e1006396. 28617870 10.1371/journal.ppat.1006396 PMC5472305

[32] Dehghan H; Oshaghi MA; Mosa-Kazemi SH; Abai MR; Rafie F; Nateghpour M; Mohammadzadeh H; Farivar L; Mohammadi Bavani M Experimental Study of *Plasmodium berghei*, *Anopheles stephensi*, and BALB/c Mouse System: Implications for Malaria Transmission Blocking Assays. Iran J. Parasitol. 2018, 13, 549–559. 30697308 PMC6348208

[33] O’Neill MJ; Bray DH; Boardman P; Chan KL; Phillipson JD; Warhurst DC; Peters W Plants as Sources of Antimalarial Drugs, Part 4: Activity of *Brucea javanica* Fruits against Chloroquine-Resistant *Plasmodium falciparum* in vitro and against *Plasmodium berghei* in vivo. J. Nat. Prod. 1987, 50, 41–48. 3298551 10.1021/np50049a007

[34] Rucci N; Ricevuto E; Ficorella C; Longo M; Perez M; Di Giacinto C; Funari A; Teti A; Migliaccio S In Vivo Bone Metastases, Osteoclastogenic Ability, and Phenotypic Characterization of Human Breast Cancer Cells. Bone 2004, 34, 697–709. 15050901 10.1016/j.bone.2003.07.012

[35] Kowashi S; Ogamino T; Kamei J; Ishikawa Y; Nishiyama S The First Total Synthesis and Absolute Stereochemistry of Plakortone G from the Jamaican Sponge *Plakortis* sp. Tetrahedron Lett. 2004, 45, 4393–4396.

[36] Jiménez-Romero C; Ortiz I; Vicente J; Vera B; Rodríguez A; Nam S; Jove R Bioactive Cycloperoxides Isolated from the Puerto Rican Sponge *Plakortis halichondrioides*. J. Nat. Prod. 2010, 73, 1694–1700. 20923180 10.1021/np100461t PMC3036788

[37] Gushiken M; Kagiyama I; Kato H; Kuwana T; Losung F; Mangindaan REP; De Voogd NJ; Tsukamoto S Manadodioxans A-E: Polyketide Endoperoxides from the Marine Sponge *Plakortis bergquistae*. J. Nat*. Med*. 2015, 69, 595–600. 26006223 10.1007/s11418-015-0920-x

[38] Meshnick SR Artemisinin: Mechanisms of Action, Resistance and Toxicity. Int. J. Parasitol. 2002, 32, 1655–1660. 12435450 10.1016/s0020-7519(02)00194-7

[39] Muregi FW; Ishih A Next-Generation Antimalarial Drugs: Hybrid Molecules as a New Strategy in Drug Design. Drug Dev. Res. 2010, 71, 20–32. 21399701 10.1002/ddr.20345 PMC3049227

[40] Daskum AM; Chessed G; Qadeer MA; Mustapha T Antimalarial Chemotherapy, Mechanisms of Action and Resistance to Major Antimalarial Drugs in Clinical Use: A Review. Microbes Infect. Dis. 2021, 2, 130–142.

[41] White NJ Review Series Antimalarial Drug Resistance. Antimalar. Drug Resist. 2004, 113, 1084–1092. 10.1172/JCI21682 PMC385418 15085184

[42] Casiolla J; Spinello A; Martini S; Bisi A; Zaffaroni N; Gobbi S Targeting Orthosteric and Allosteric Pockets of Aromatase via Dual-Mode Novel Azole Inhibitors. *ACS Med*. Chem. Lett. 2020, 11, 732–739. 10.1021/acsmedchemlett.9b00591 PMC7236249 32435378

[43] Fahy E; Potts B; Faulkner J 6-Bromotryptamine Derivatives from the Gulf of California Tunicate *Didemnum candidum*. J. Nat. Prod. 1991, 54, 564–569.

[44] Chantana C; Sirion U; Iawsipo P; Jaratjaroonphong J Short Total Synthesis of (±)-Gelliusine e and 2,3′-Bis(Indolyl)Ethylamines via PTSA-Catalyzed Transindolylation. J. Org. Chem. 2021, 86, 13360–13370. 34528793 10.1021/acs.joc.1c01461

[45] Hussain A; Bourguet-Kondracki ML; Majeed M; Ibrahim M; Imran M; Yang XW; Ahmed I; Altaf AA; Khalil AA; Rauf A; Marine Life as a Source for Breast Cancer Treatment: A Comprehensive Review. Biomed. Pharmacother. 2023, 159, 114165. 36634590 10.1016/j.biopha.2022.114165

[46] Spinello A; Martini S; Berti F; Pennati M; Pavlin M; Sgrignani J; Grazioso G; Colombo G; Zaffaroni N; Magistrato A Rational Design of Allosteric Modulators of the Aromatase Enzyme: An Unprecedented Therapeutic Strategy to Fight Breast Cancer. Eur. J. Med. Chem. 2019, 168, 253–262. 30822713 10.1016/j.ejmech.2019.02.045

[47] Lau YS; Danks L; Sun SG; Fox S; Sabokbar A; Harris A; Athanasou NA RANKL-Dependent and RANKL-Independent Mechanisms of Macrophage-Osteoclast Differentiation in Breast Cancer. Breast Cancer Res. Treat. 2007, 105, 7–16. 10.1007/s10549-006-9438-y 17151927

[48] Kang H; Xiao X; Huang C; Yuan Y; Tang D; Dai X; Zeng X Potent Aromatase Inhibitors and Molecular Mechanism of Inhibitory Action. Eur. J. Med. Chem. 2018, 143, 426–437. 29202405 10.1016/j.ejmech.2017.11.057

[49] Norris MD; Perkins MV; Sorensen EJ Biomimetic Total Synthesis of Gracilioethers B and C. Org. Lett. 2015, 17, 668–671. 25621375 10.1021/ol503695j

[50] Di Micco S; Zampella A; D’Auria MV; Festa C; De Marino S; Riccio R; Butts CP; Bifulco G Plakilactones G and H from a Marine Sponge. Stereochemical Determination of Highly Flexible Systems by Quantitative NMR-Derived Interproton Distances Combined with Quantum Mechanical Calculations of 13C Chemical Shifts. Beilstein J. Org. Chem 2013, 9, 2940–2949. 24454574 10.3762/bjoc.9.331 PMC3896268

