# Supplementary material for: Two Gracilioethers Containing a [2(5H)-Furanylidene]ethanoate Moiety and 9,10-Dihydroplakortone G: New Polyketides from the Caribbean Marine Sponge Plakortis halichondrioides
Source: Appl Sci (Basel). Author manuscript; Available in PMC 2024 Dec 30. (PMC11684765; doi:10.3390/app14010281)
Supplement: Supp [file NIHMS2000060-supplement-Supp.pdf]

Two Gracilioethers Containing a [2(5H)-Furanylidene]ethanoate Moiety and 9,10-Dihydroplakortone G: New Polyketides from the Caribbean Marine Sponge *Plakortis halichondrioides*

Luis A. Amador 1, Abimael D. Rodríguez 1,*, Lesly Carmona-Sarabia 1, Emilee E. Colón-Lorenzo 2 and Adelfa E. Serrano 2

1 Molecular Sciences Research Center, University of Puerto Rico, 1390 Ponce de León Avenue, San Juan 00926, Puerto Rico; luisalberto.amador@upr.edu (L.A.A.); lesly.carmona@upr.edu (L.C.-S.)
2 Department of Microbiology and Medical Zoology, University of Puerto Rico School of Medicine, San Juan 00921, Puerto Rico; emilee.colon@upr.edu (E.E.C.-L.); adelfa.serrano@upr.edu (A.E.S.)

* Corresponding author. Tel.: +1-787-523-5320; fax: +1-787-522-2150; e-mail:abimael.rodriguez1@upr.edu

Supplementary Material

Table S1. NMR spectral data for plakortone G (**9**) in $CDCl_3$.

atom	δ_C[a], Type	δ_H[b], mult (*J* in Hz)	^{1}H-^{1}H COSY	^{1}H-^{13}C HMBC
1	173.5, C			
2	135.8, C			
3	150.1, CH	6.82, br s		1, 2, 4, 13
4	89.3, C			
5	37.2, CH_2	1.60, m		3, 4, 15, 16
		1.60, m		3, 4, 15, 16
6	21.2, CH_2	1.10-1.38, br m		
7	35.1, CH_2	1.10-1.38, br m		8
		1.10-1.38, br m		8
8	44.3, CH	1.73, m	9	
9	132.9, CH	5.01, dd (8.9, 15.2)	8, 10	8
10	132.4, CH	5.34, m	9, 11	8
11	25.6, CH_2	1.97, m	10, 12	9, 10, 12
12	14.2, CH_3	0.94, t (7.5)	11	10, 11
13	18.5, CH_2	2.28, m	14	1, 2, 3, 14
14	12.0, CH_3	1.15, t (7.5)	13	2, 13
15	29.9, CH_2	1.70, m	16	3, 4, 5, 16
15	29.9, CH_2	1.70, m	16	3, 4, 5, 16
16	7.7, CH_3	0.80, m	15α, 15β	4, 5, 15
17	28.2, CH_2	1.10-1.38, br m		8
		1.10-1.38, br m		8
18	11.6, CH_3	0.80, m		8

*** All Assignments are based on COSY, HSQC, and HMBC experiments. [a] Recorded at 125 MHz. Multiplicities were obtained from the Attached Proton Test (APT) experiments. [b] Recorded at 500 MHz.**

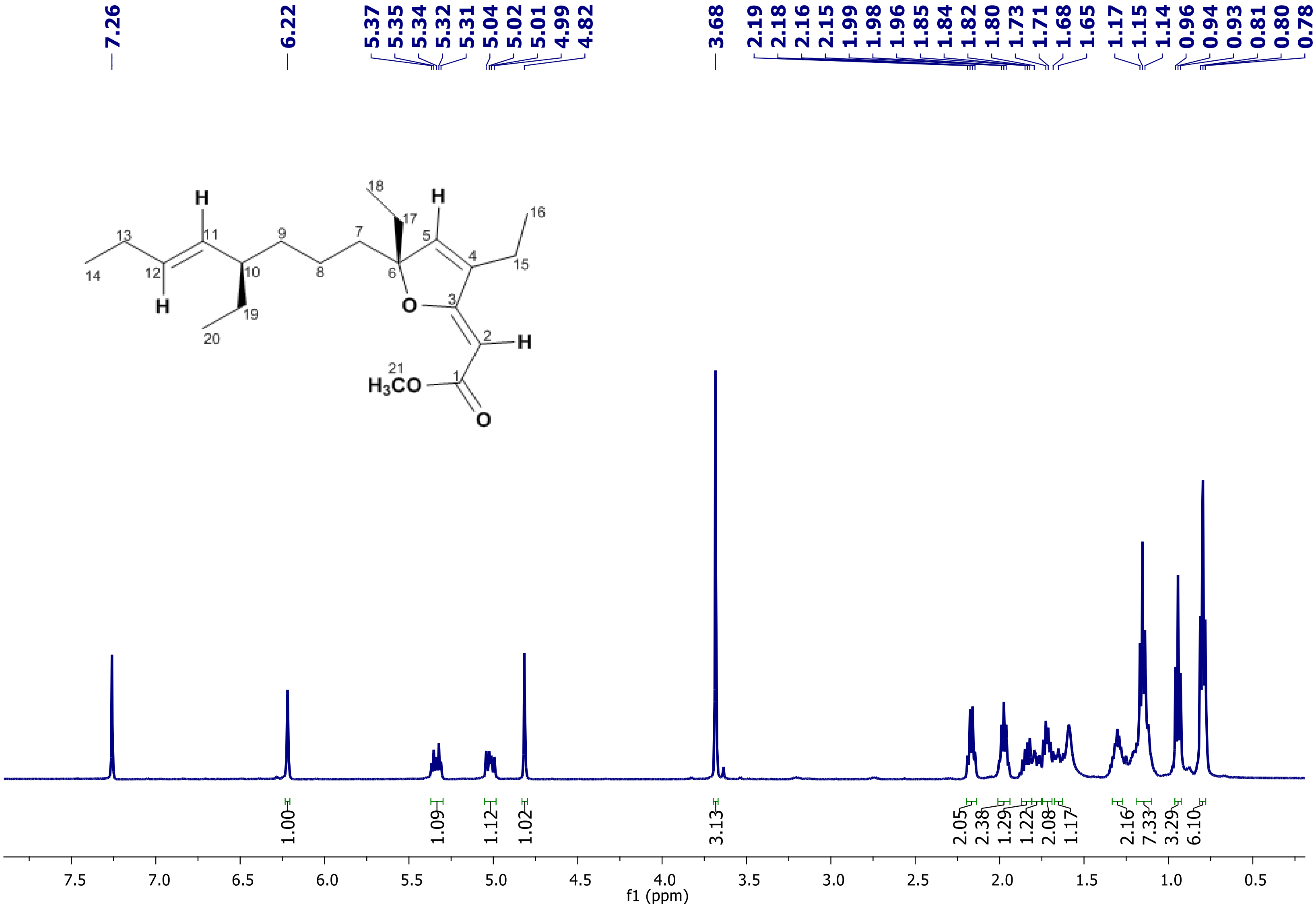

Figure S1. ^{1}H-NMR spectrum ($CDCl_3$, 500 MHz) of gracilioether M (**6**)

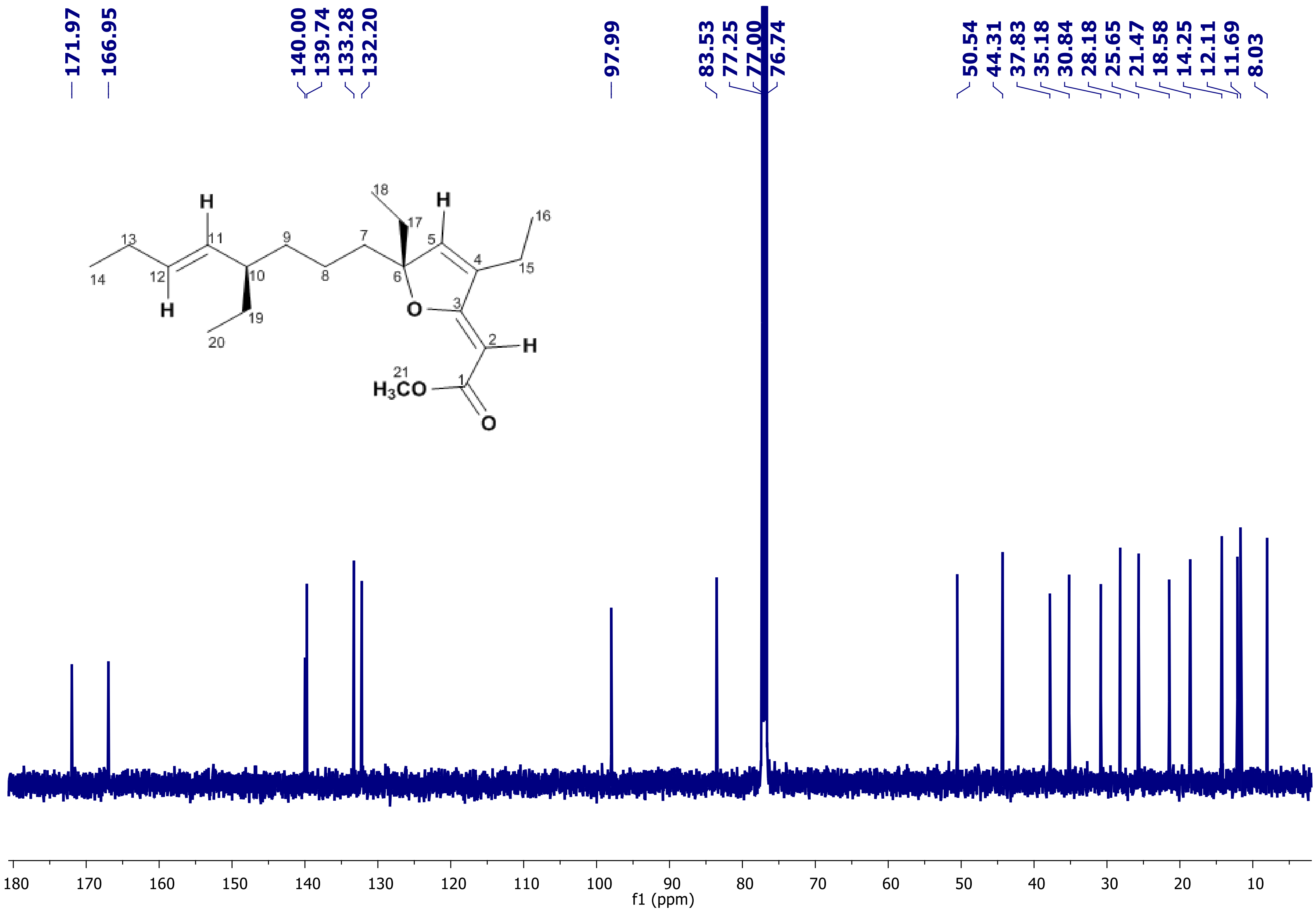

Figure S2. ^{13}C-NMR spectrum ($CDCl_3$, 125 MHz) of gracilioether M (**6**).

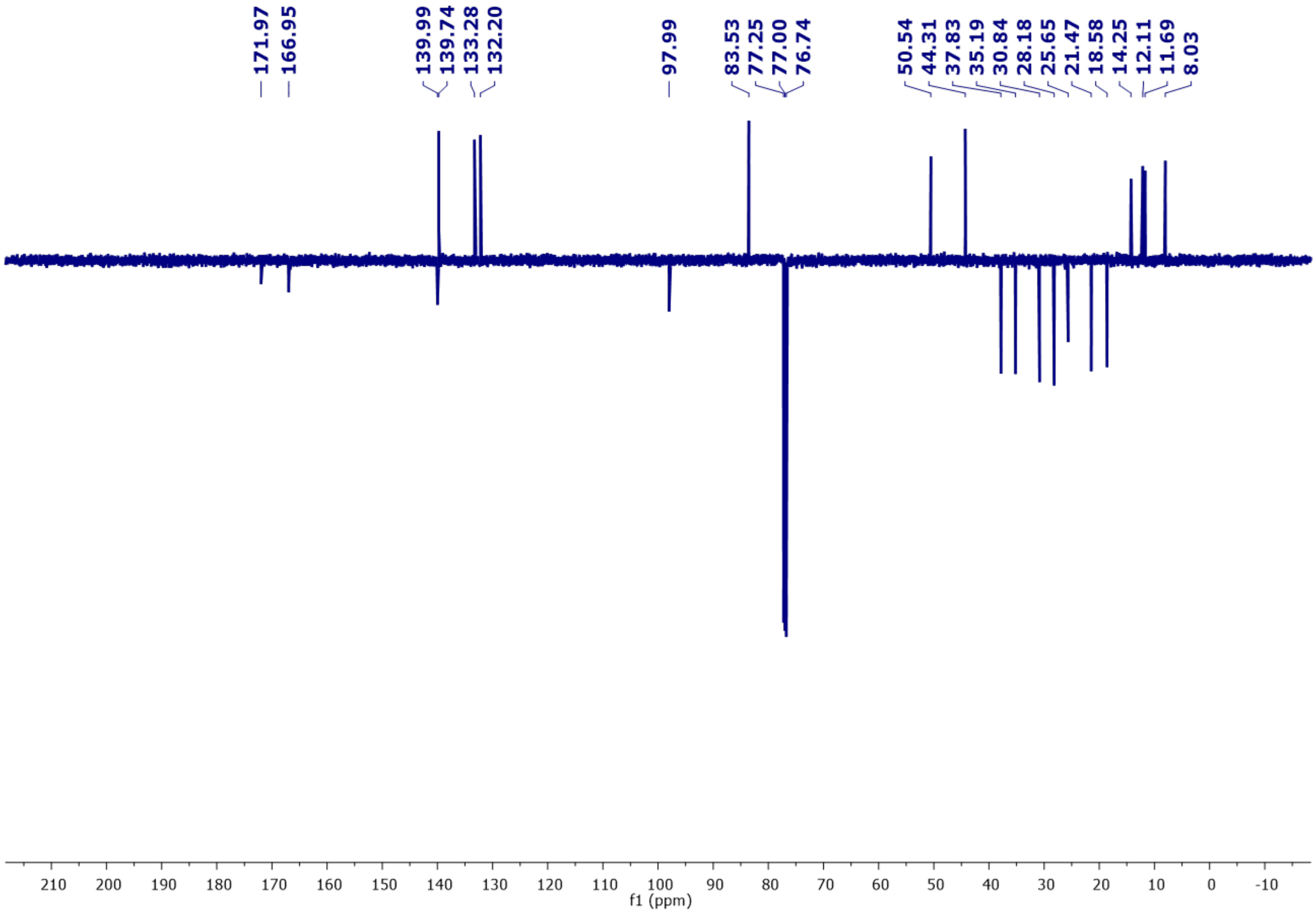

Figure S3. APT spectrum ($CDCl_3$, 125 MHz) of gracilioether M (**6**).

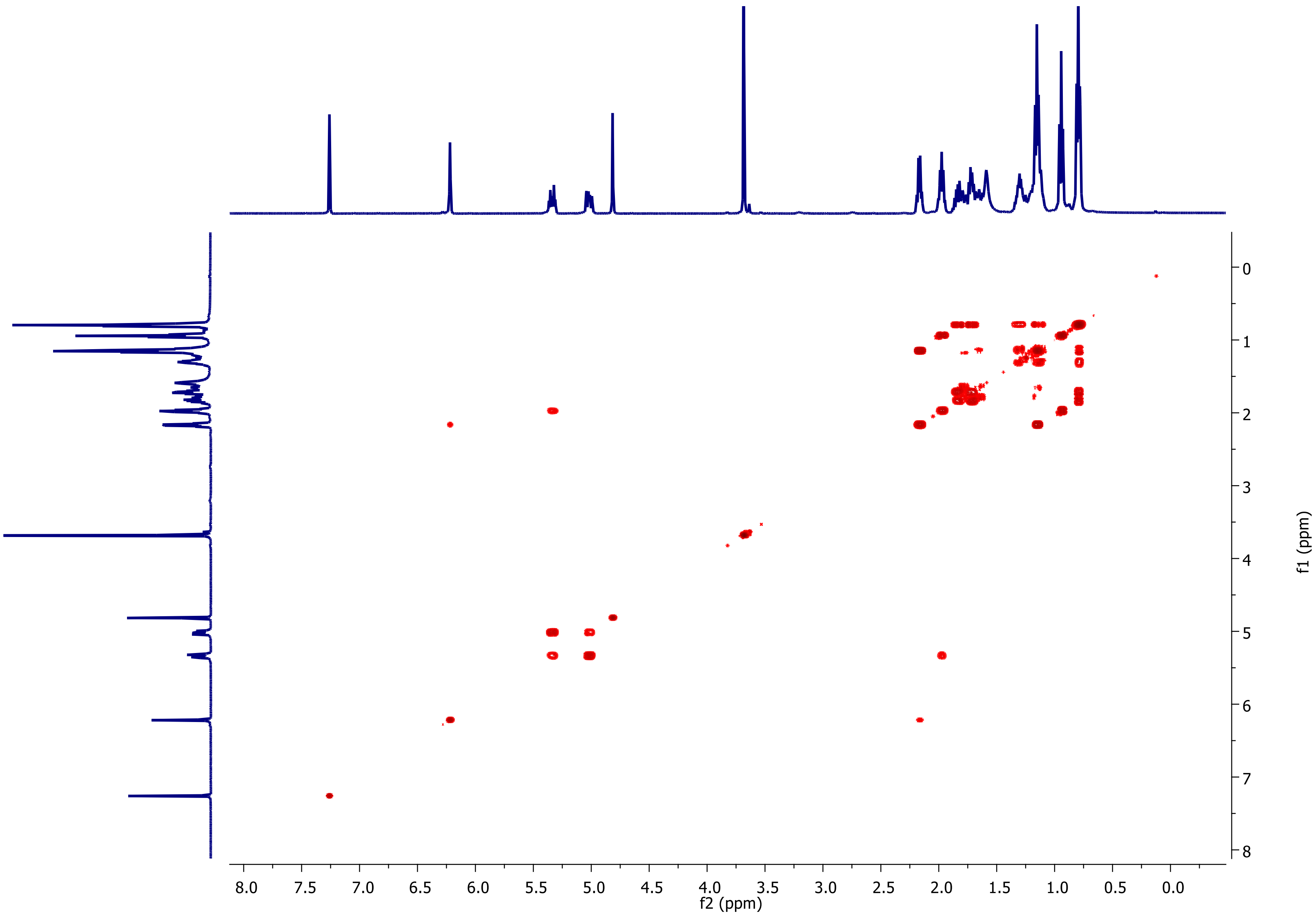

Figure S4. ^{1}H-^{1}H-COSY spectrum ($CDCl_3$) of gracilioether M (**6**).

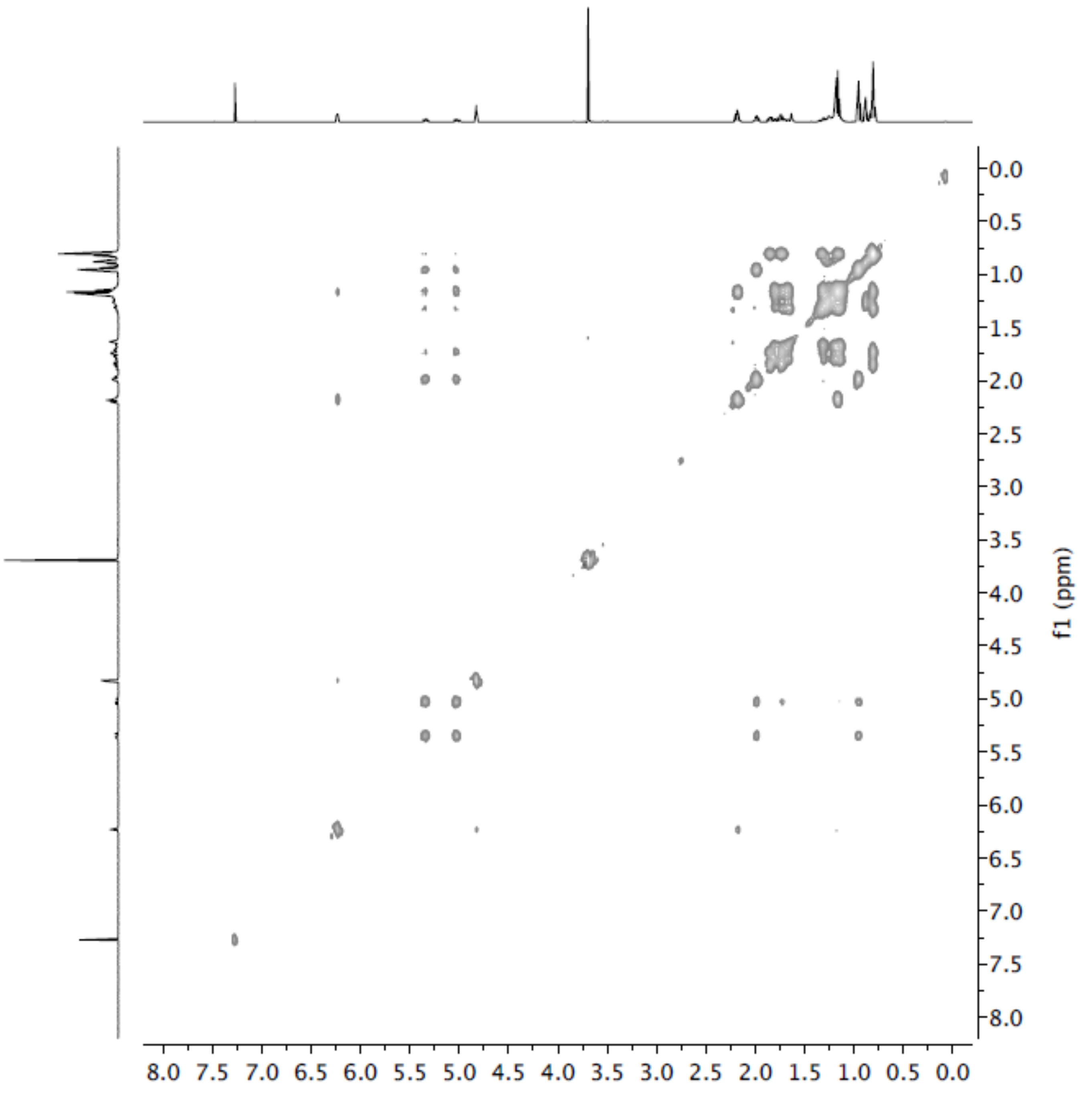

Figure S5. ^{1}H-^{1}H-TOCSY spectrum ($CDCl_3$) of gracilioether M (**6**)

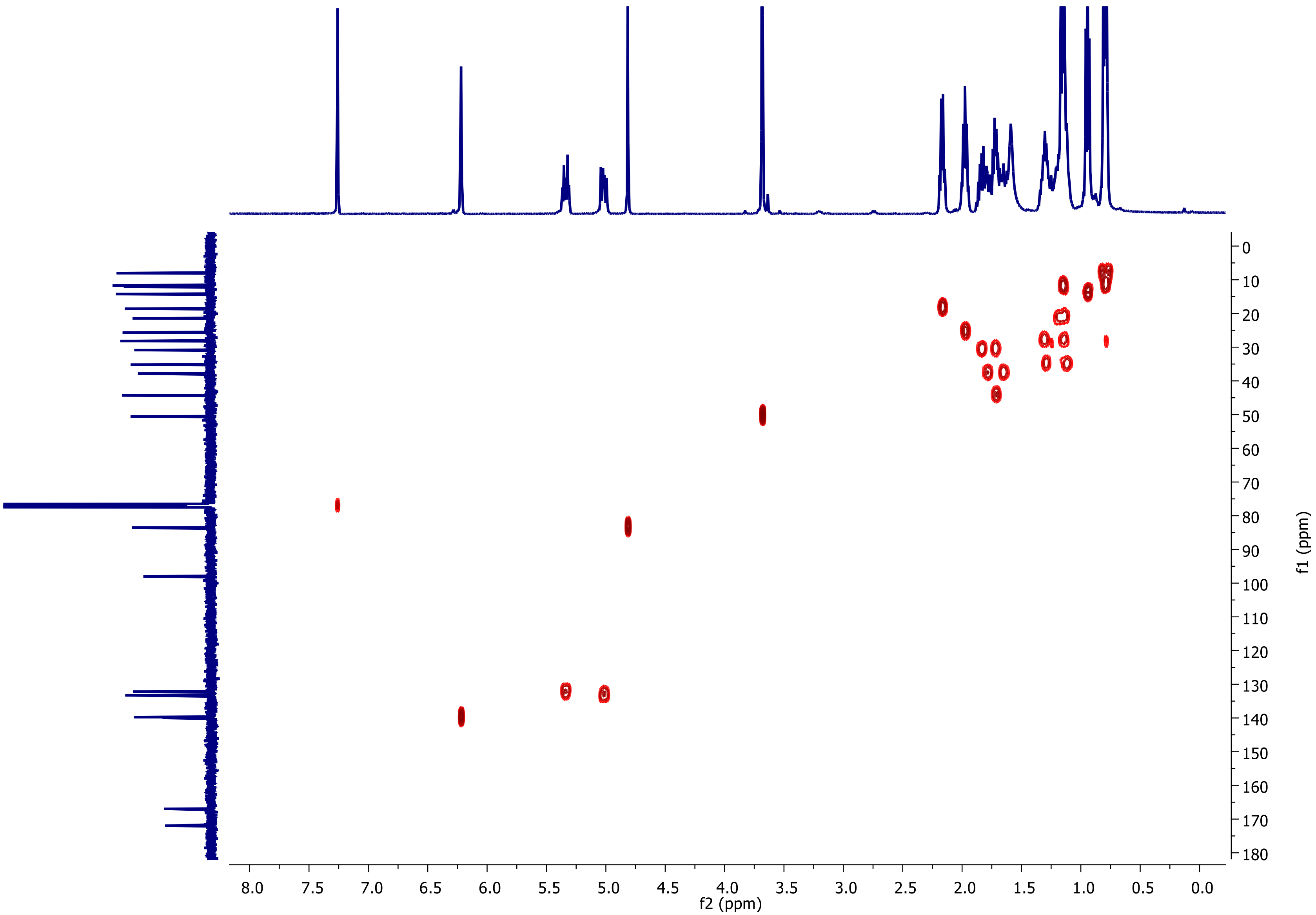

Figure S6. ^{1}H-^{13}C-HSQC spectrum ($CDCl_3$) of gracilioether M (**6**).

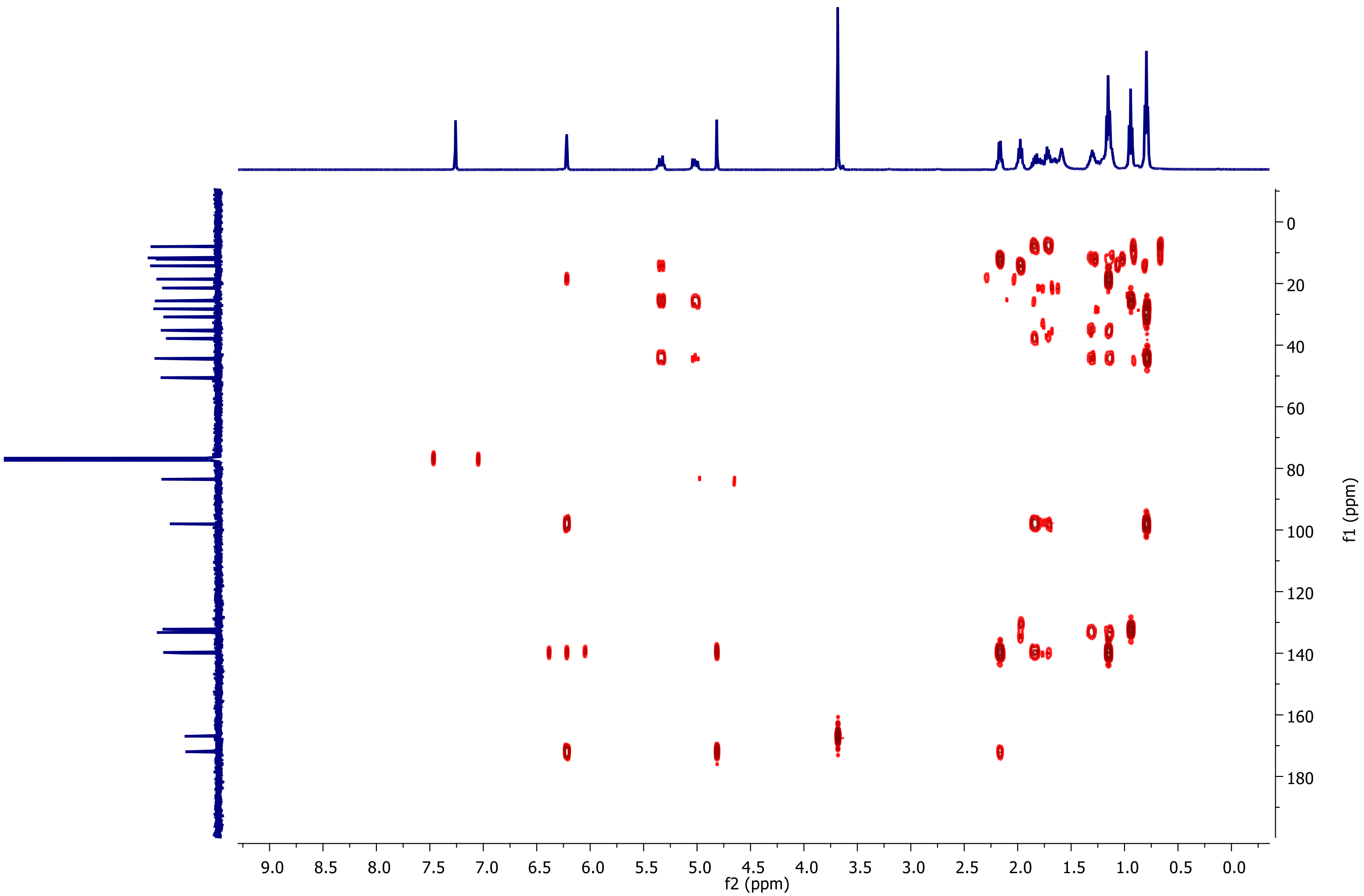

Figure S7. ^{1}H-^{13}C-HMBC spectrum ($CDCl_3$) of gracilioether M (**6**).

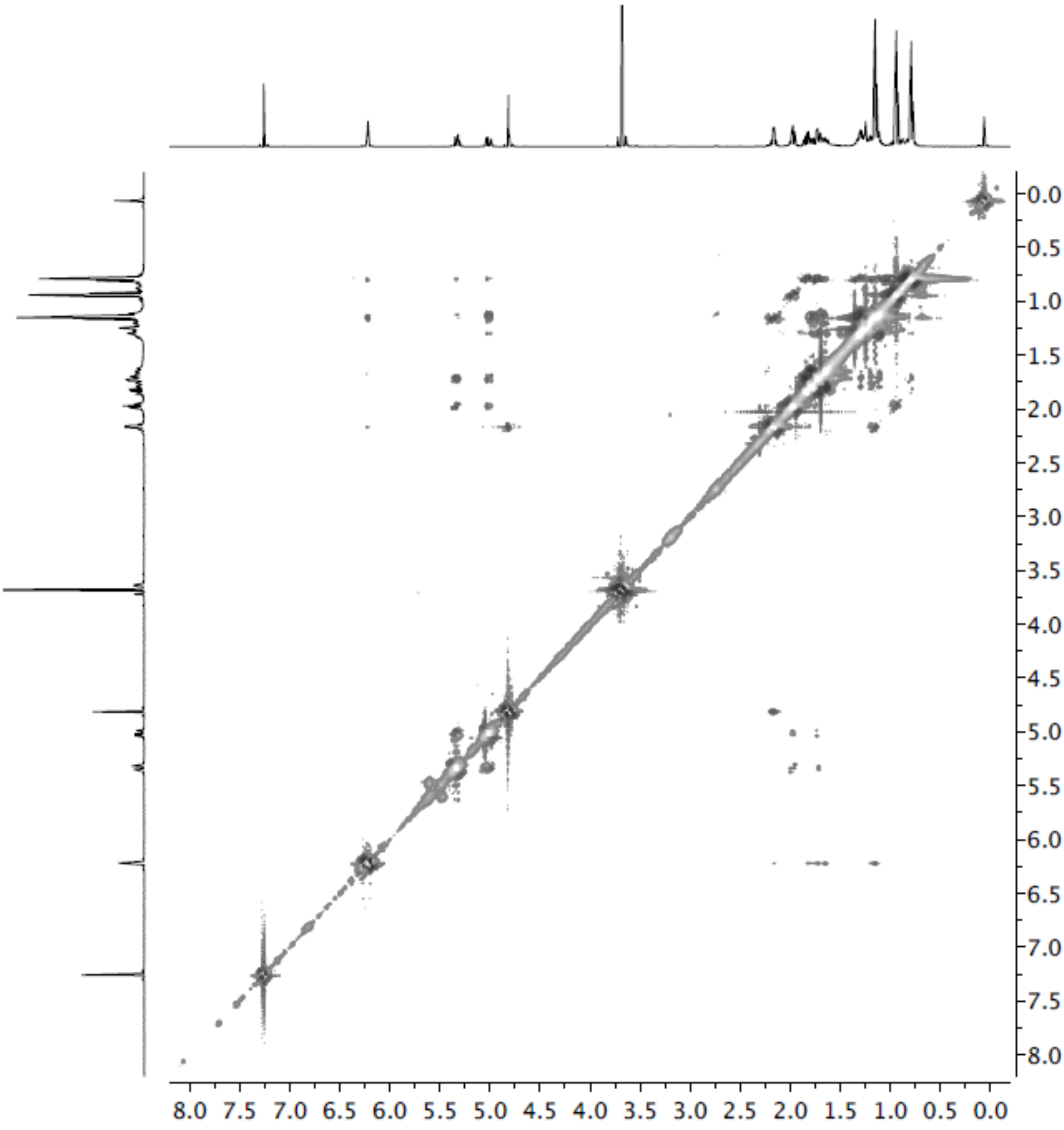

Figure S8. ^{1}H-^{1}H-NOESY spectrum ($CDCl_3$) of gracilioether M (**6**).

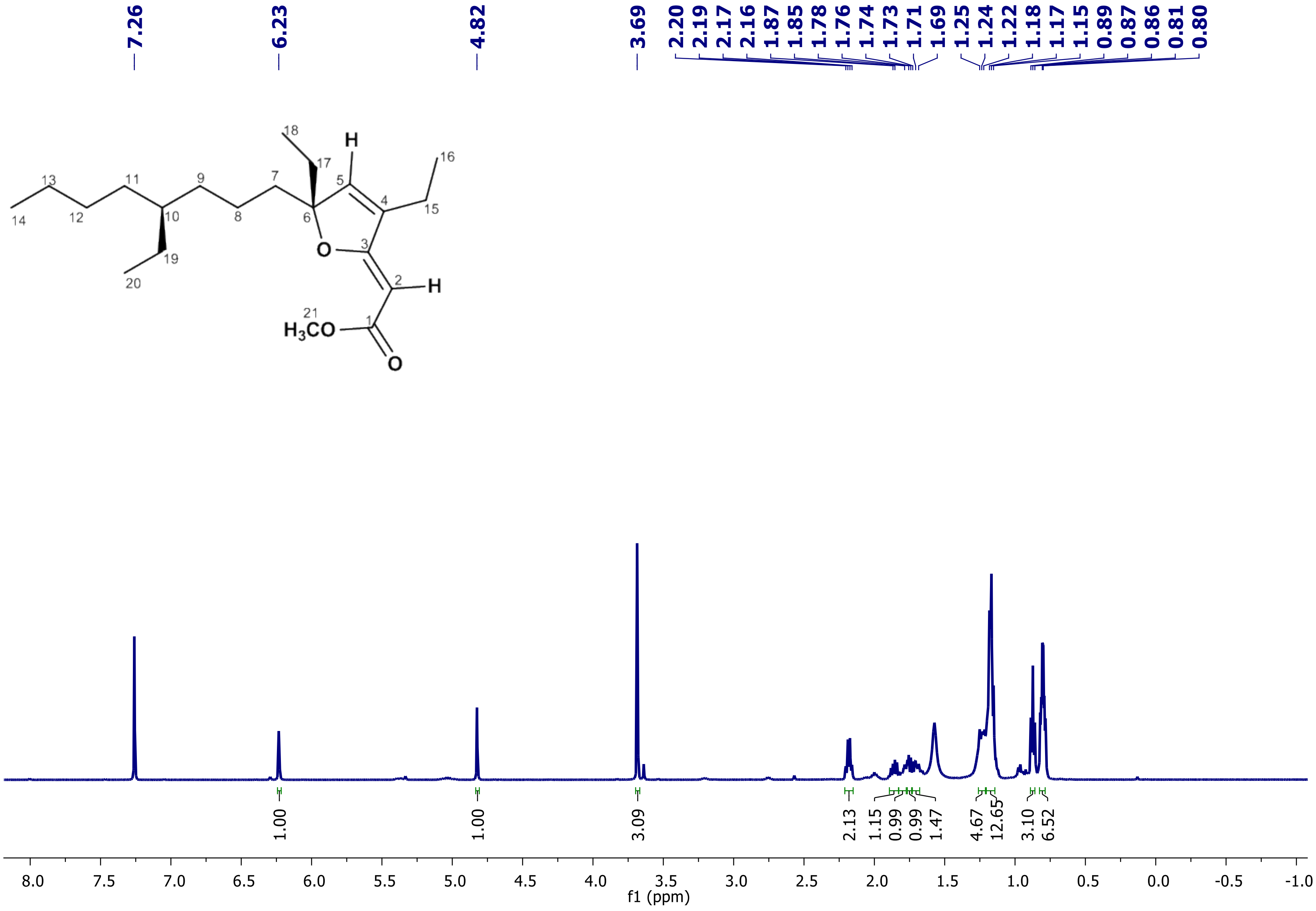

Figure S9. ^{1}H-NMR spectrum ($CDCl_3$, 500 MHz) of 11,12-dihydrogracilioether M (**7**).

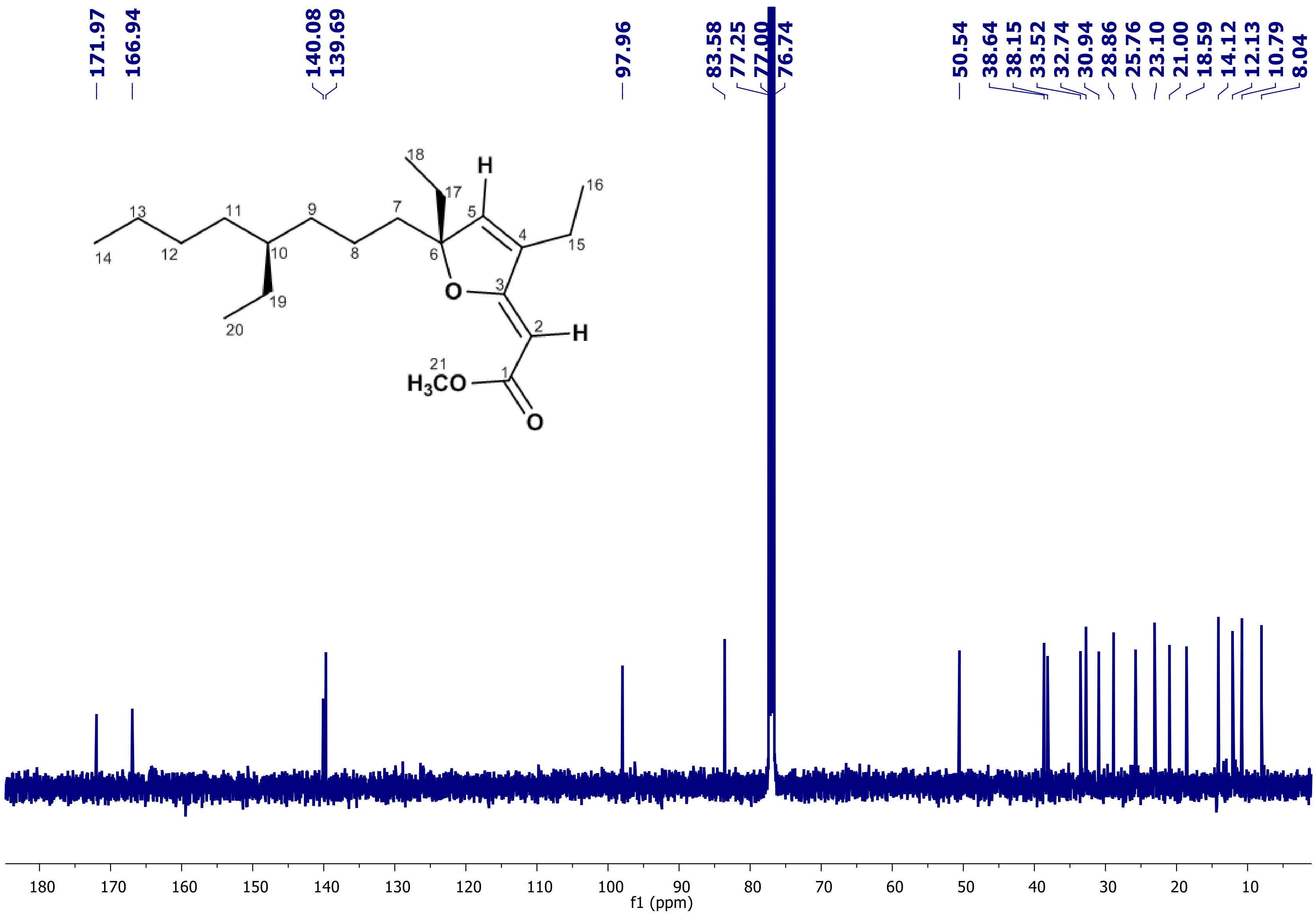

Figure S10. ^{13}C-NMR spectrum ($CDCl_3$, 125 MHz) of 11,12-dihydrogracilioether M (**7**).

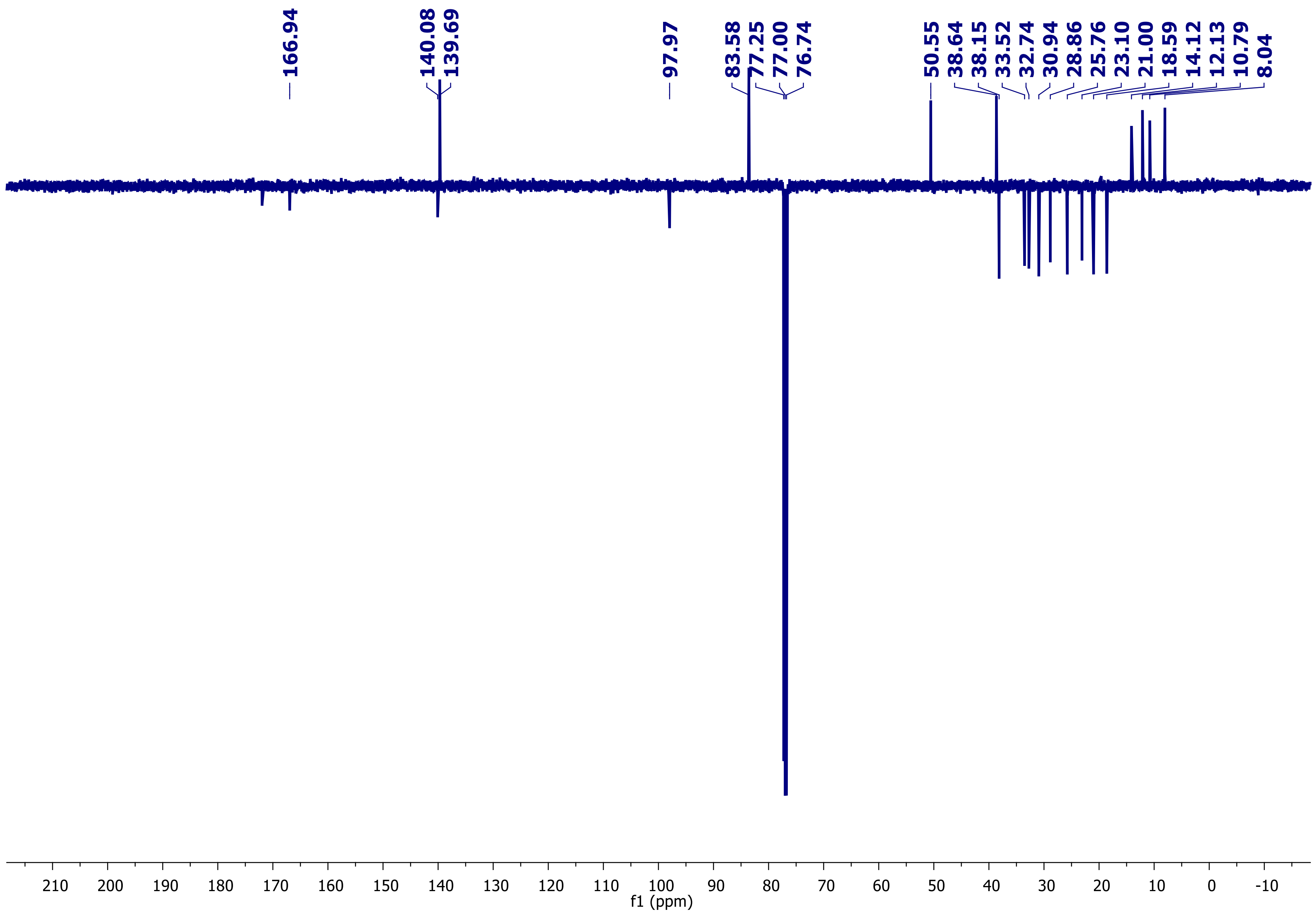

Figure S11. APT spectrum ($CDCl_3$, 125 MHz) of 11,12-dihydrogracilioether M (**7**).

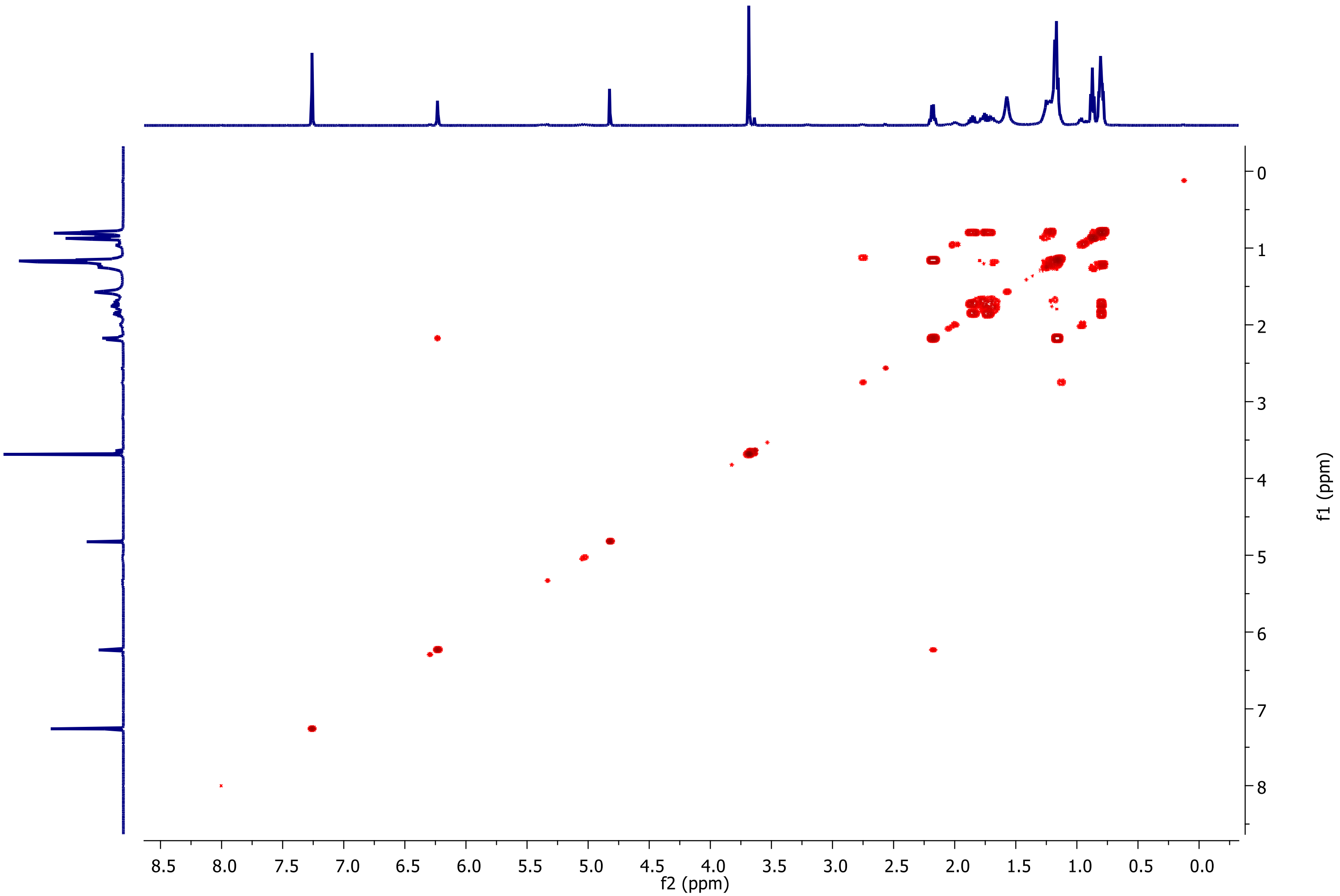

Figure S12. ^{1}H-^{1}H-COSY spectrum ($CDCl_3$) of 11,12-dihydrogracilioether M (**7**).

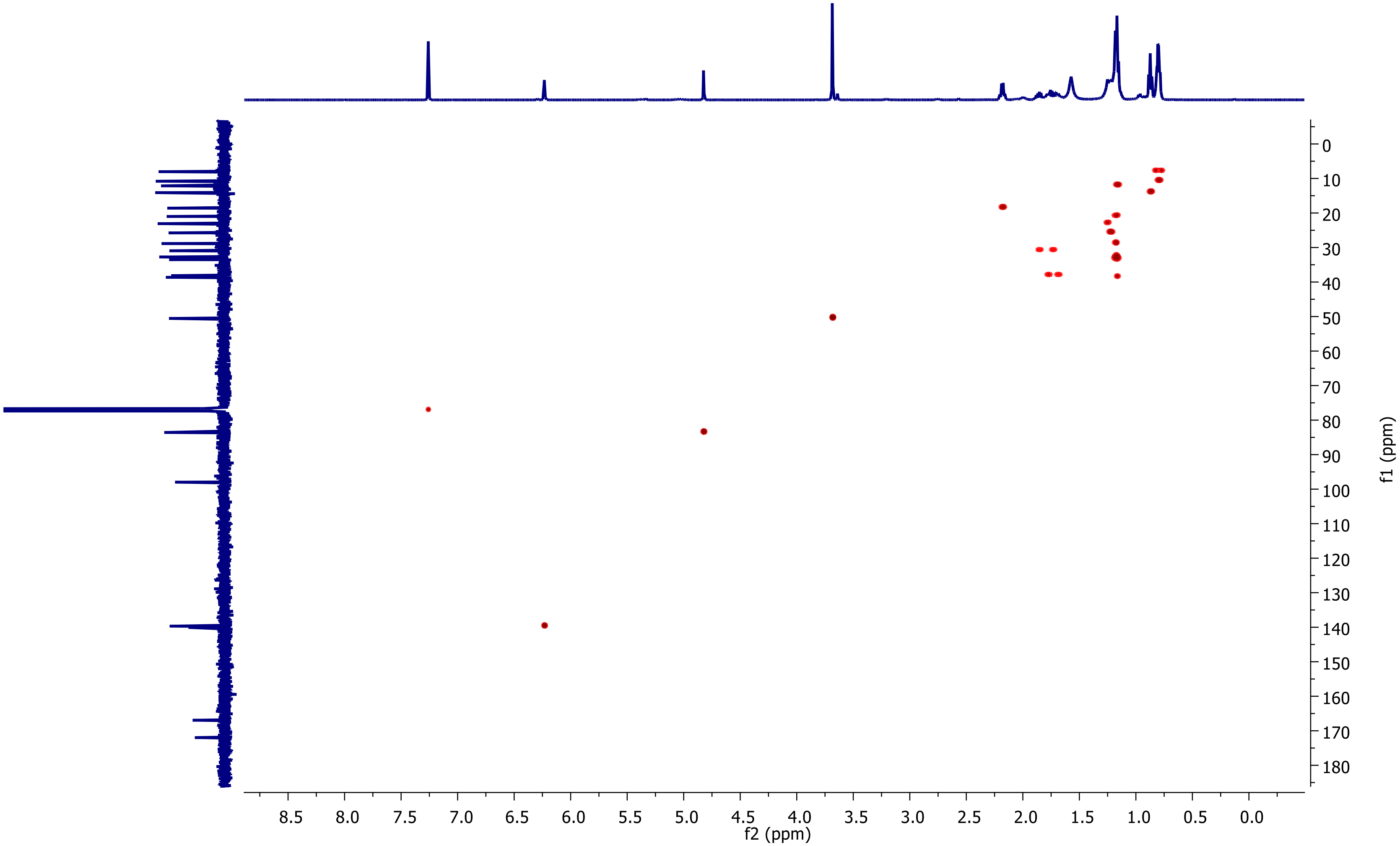

Figure S13. ^{1}H-^{13}C-HSQC spectrum ($CDCl_3$) of 11,12-dihydrogracilioether M (**7**).

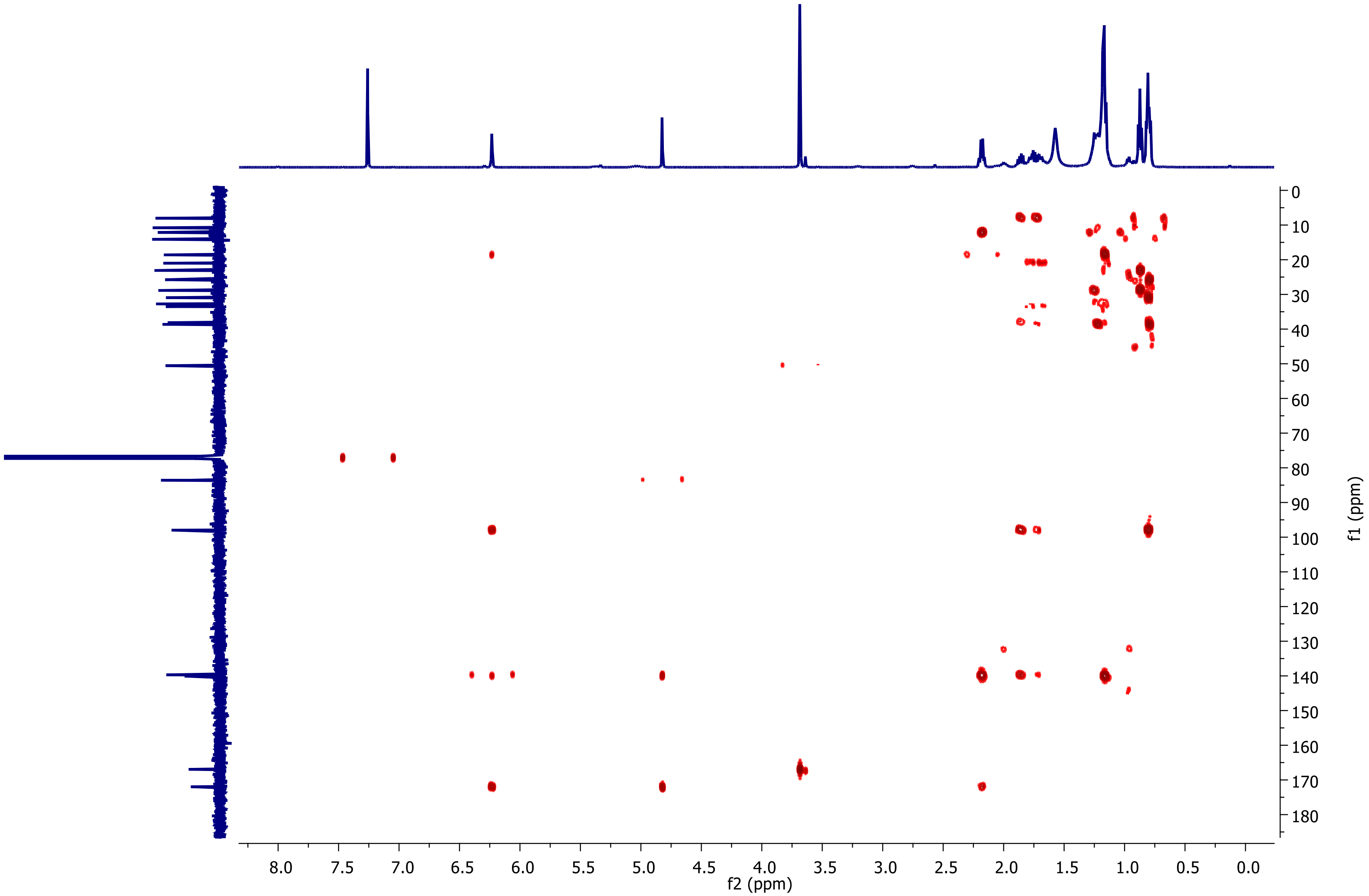

Figure S14. ^{1}H-^{13}C-HMBC spectrum ($CDCl_3$) of 11,12-dihydrogracilioether M (**7**).

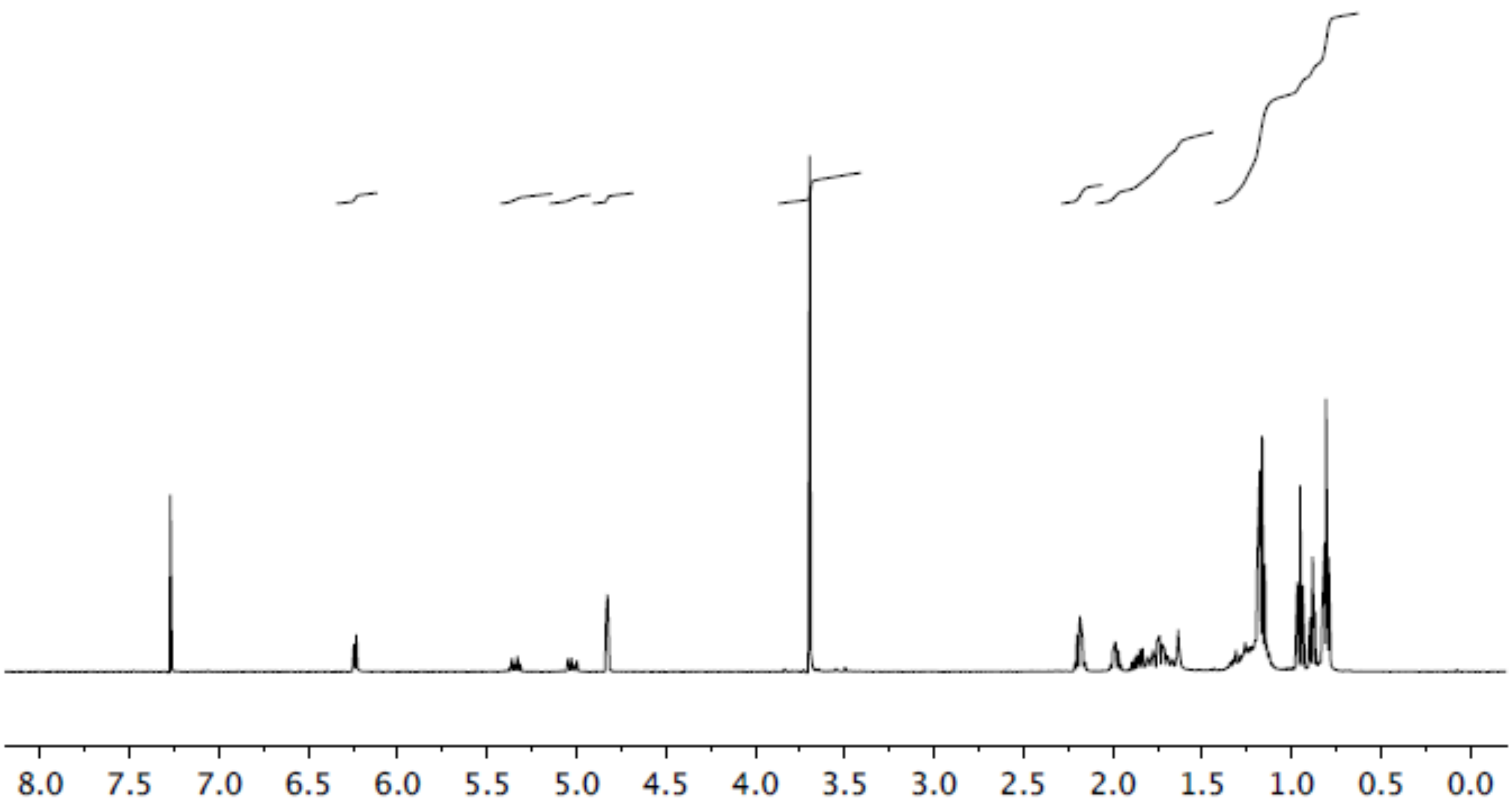

Figure S15. ^{1}H-NMR spectrum ($CDCl_3$, 500 MHz) of a mixture of compounds **8**–**9**.

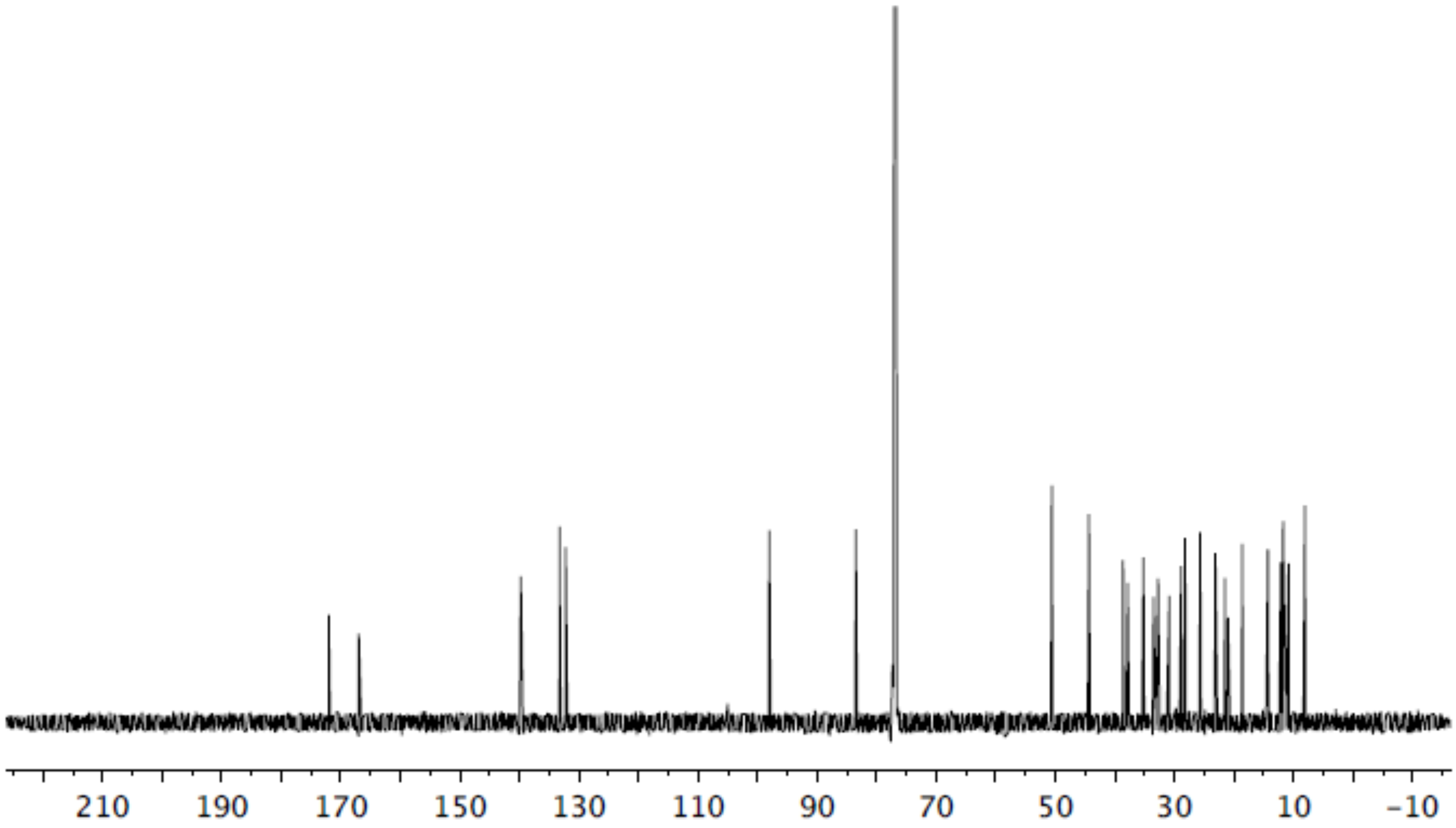

Figure S16. ^{13}C-NMR spectrum ($CDCl_3$, 125 MHz) of a mixture of compounds **8**–**9**.

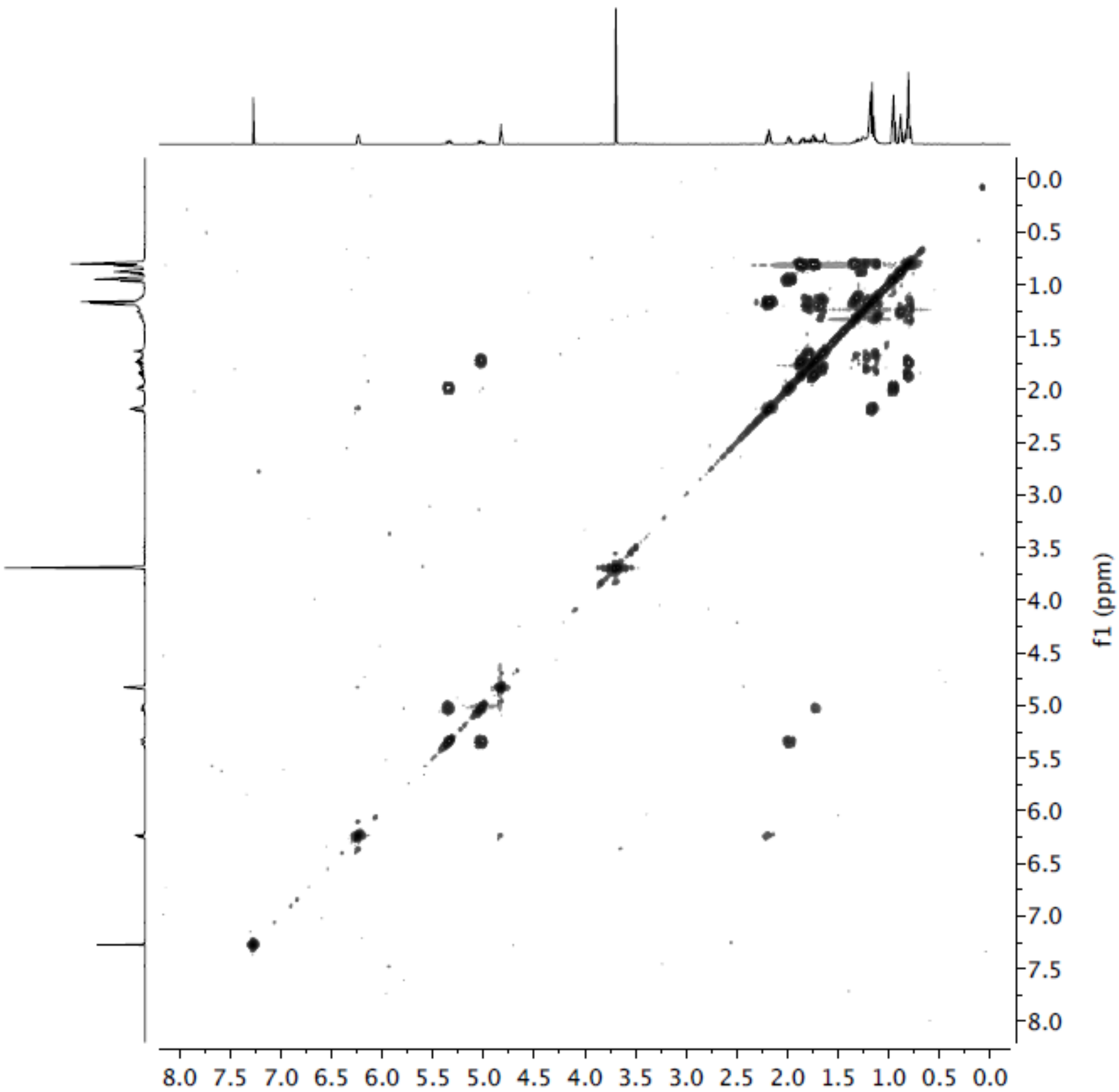

Figure S17. ^{1}H-^{1}H-COSY spectrum ($CDCl_3$) of a mixture of compounds **8**–**9**.

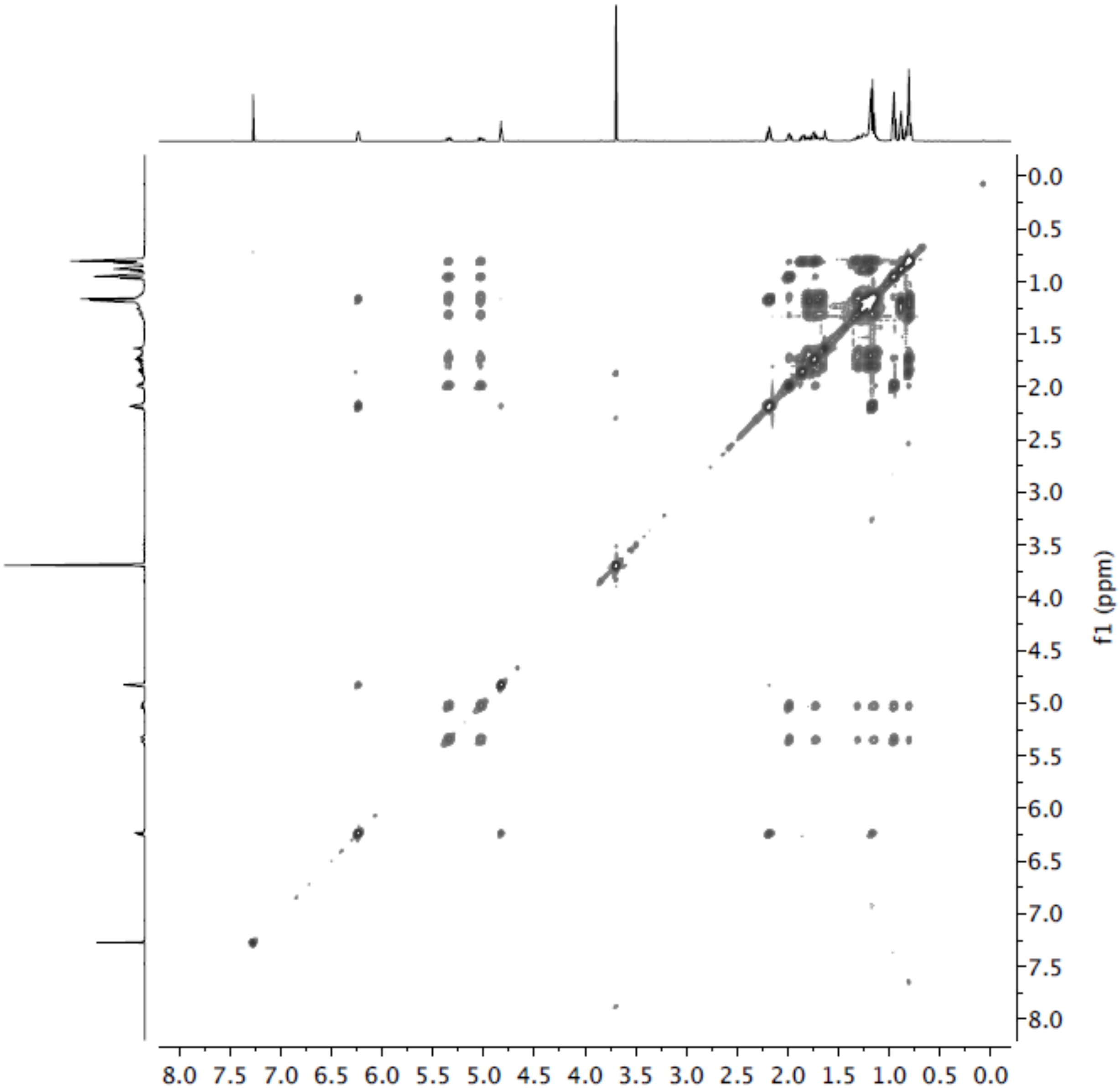

Figure S18. ^{1}H-^{1}H-TOCSY spectrum ($CDCl_3$) of a mixture of compounds **8**–**9**.

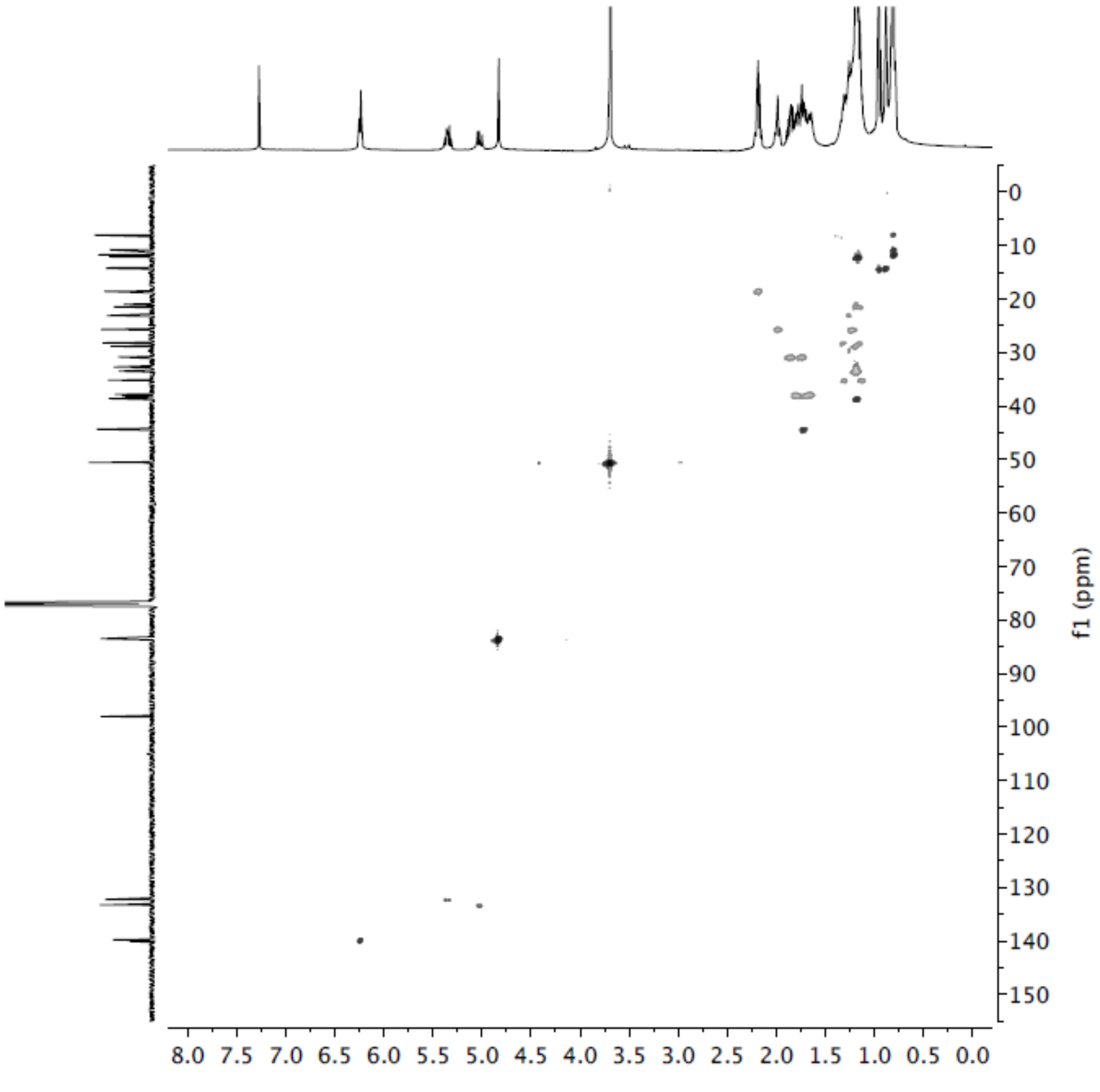

Figure S19. ^{1}H-^{13}C-HSQC spectrum ($CDCl_3$) of a mixture of compounds **8**–**9**.

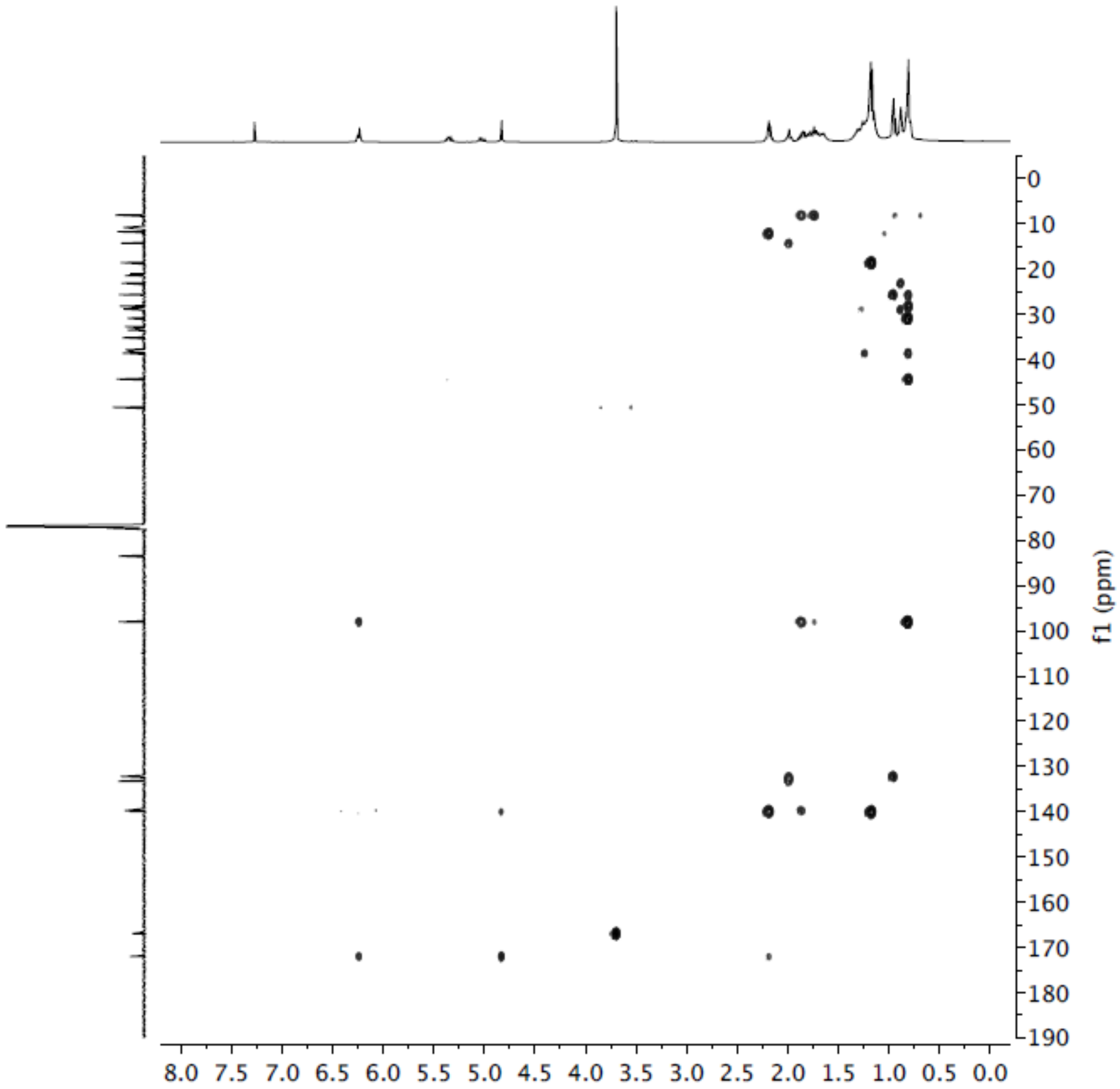

Figure S20. ^{1}H-^{13}C-HMBC spectrum ($CDCl_3$) of a mixture of compounds **8**–**9**.

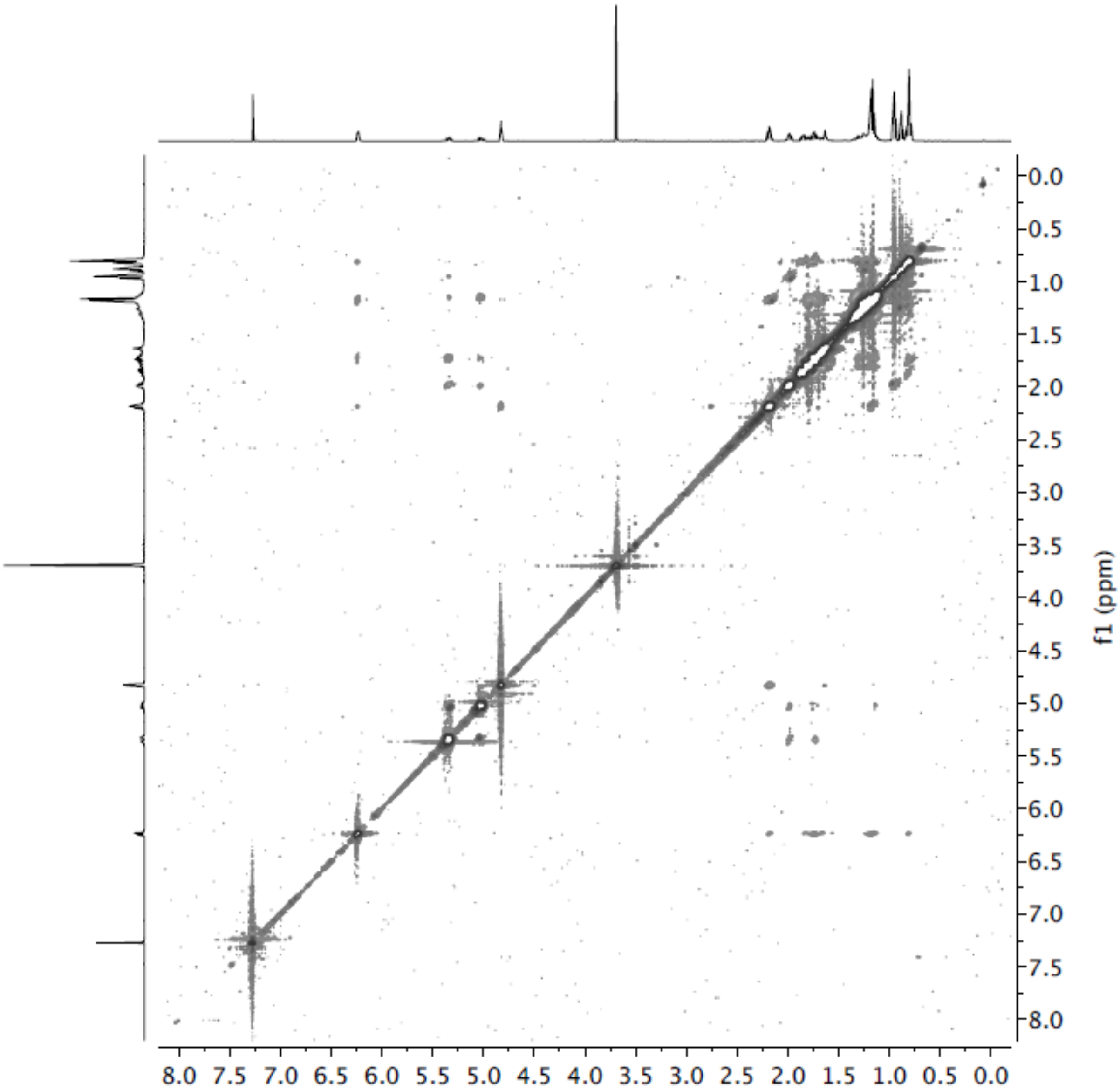

Figure S21. ^1H-^1H-NOESY spectrum ($CDCl_3$) of a mixture of compounds **8–9**.

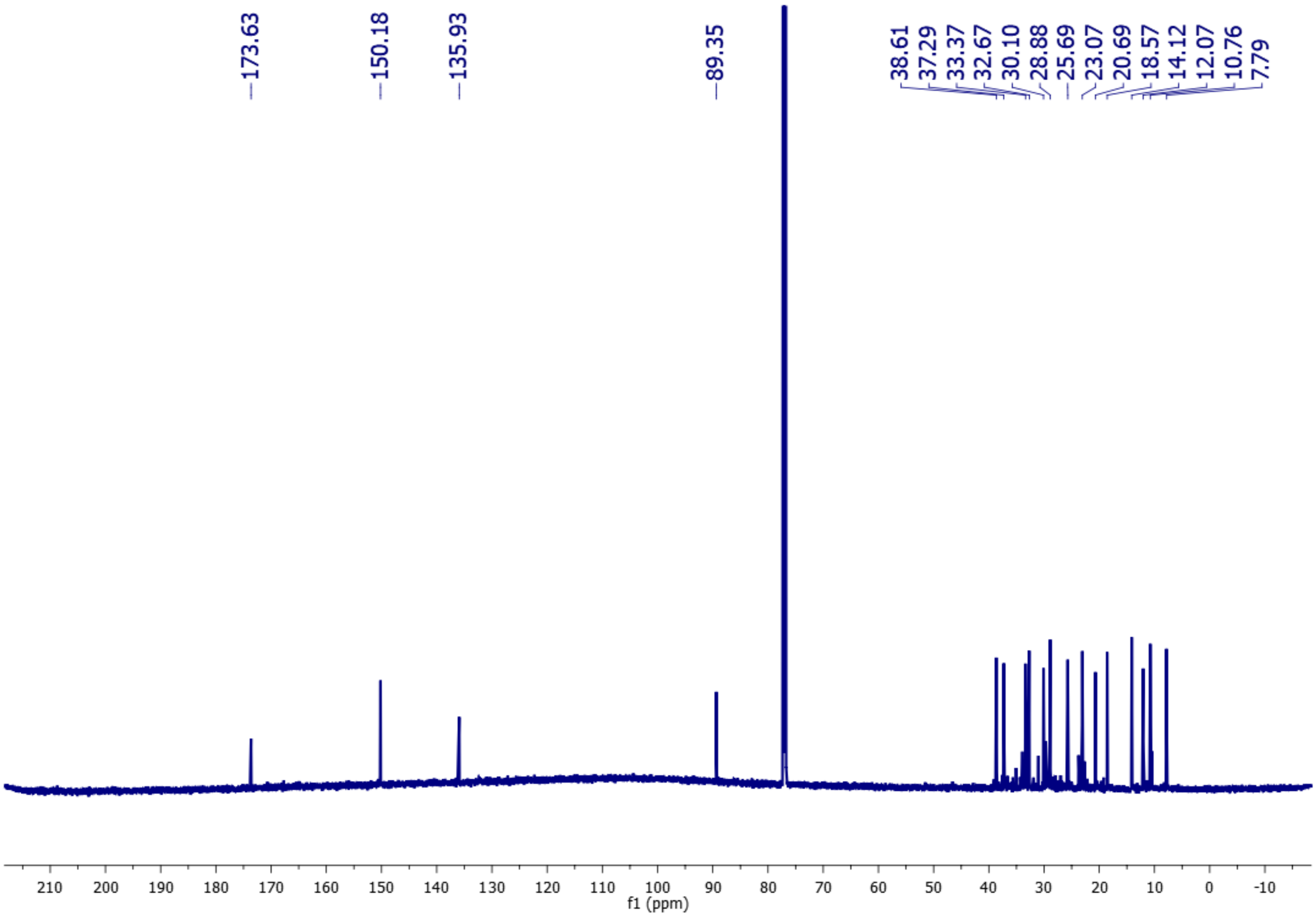

Figure S22. ^{13}C-NMR spectrum ($CDCl_3$, 125 MHz) for semi-synthetic 9,10-dihydroplakortone G (**8**) (~90% pure).

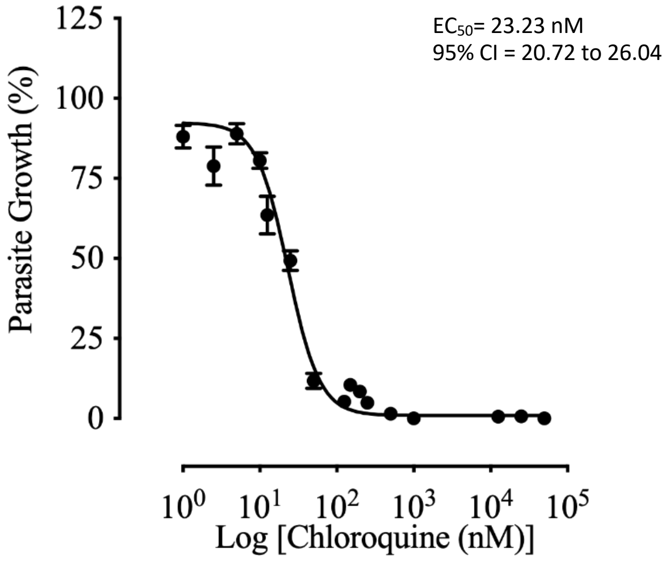

Figure S23. Dose-response curve of the parasite *Plasmodium berghei* for chloroquine (positive control).

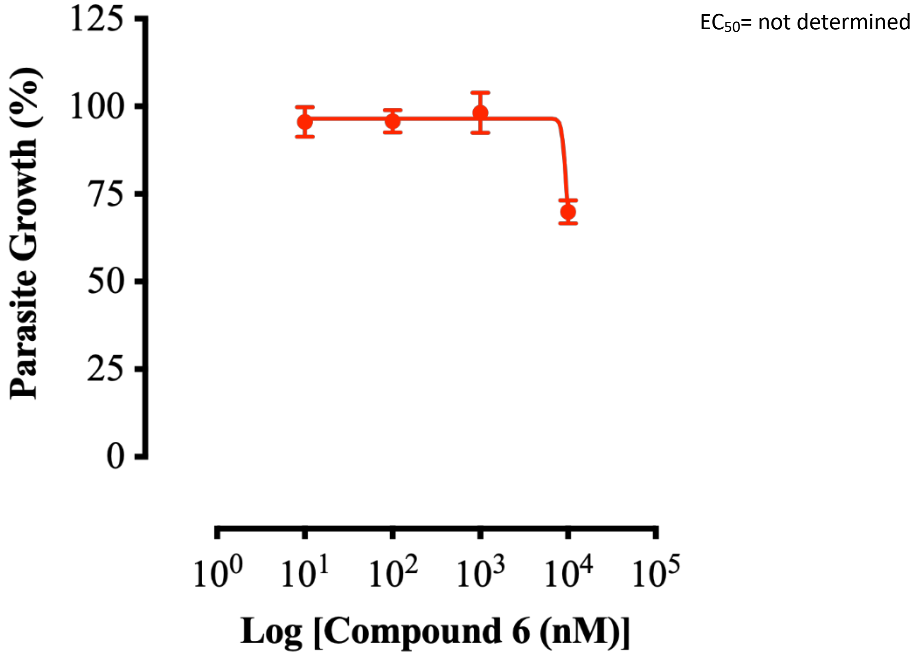

Figure S24. Dose-response curve of the parasite *Plasmodium berghei* for gracilioether M (**6**)

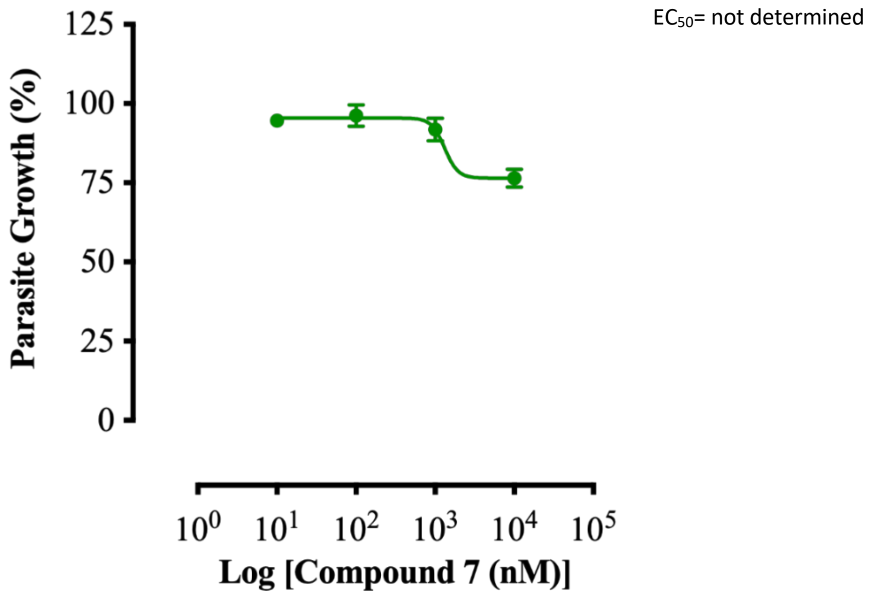

Figure S25. Dose-response curve of the parasite *Plasmodium berghei* for 11,12-dihydrogracilioether M (**7**).

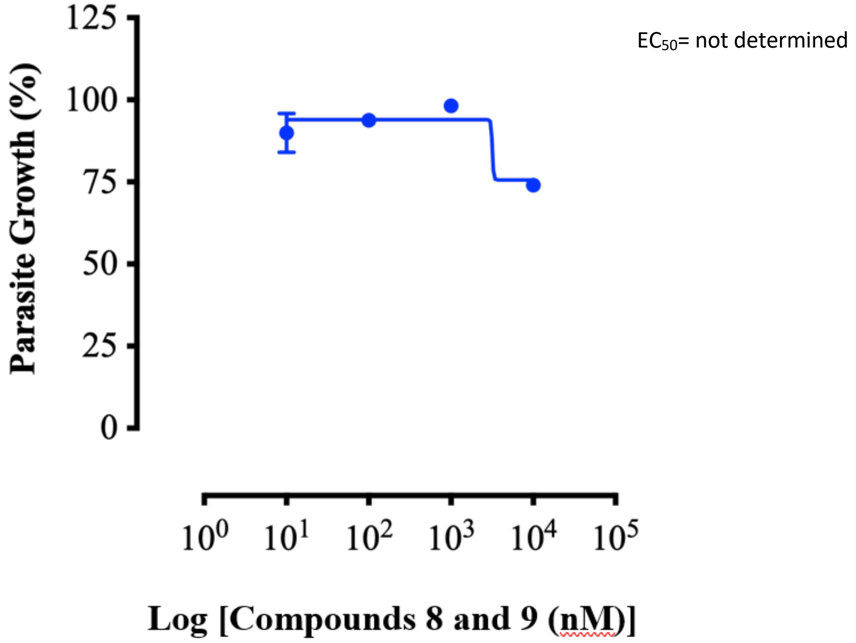

Figure S26. Dose-response curve of the parasite *Plasmodium berghei* for a mixture of compounds **8**–**9**
